# Take care of the environment: housing conditions affect the interplay of nutritional interventions and intestinal microbiota in broiler chickens

**DOI:** 10.1186/s42523-019-0009-z

**Published:** 2019-08-27

**Authors:** Jannigje G. Kers, Francisca C. Velkers, Egil A. J. Fischer, Gerben D. A. Hermes, David M. Lamot, J. Arjan Stegeman, Hauke Smidt

**Affiliations:** 1 Faculty of Veterinary Medicine, Department of Farm Animal Health, Utrecht University, Utrecht, the Netherlands; 2 Laboratory of Microbiology, Wageningen University and Research, Wageningen, the Netherlands; 3 Cargill Animal Nutrition Innovation Center, Velddriel, the Netherlands

**Keywords:** Transmission, Microbiome, 16S rRNA, Fatty acids, Poultry

## Abstract

**Background:**

The intestinal microbiota is shaped by many interactions between microorganisms, host, diet, and the environment. Exposure to microorganisms present in the environment, and exchange of microorganisms between hosts sharing the same environment, can influence intestinal microbiota of individuals, but how this affects microbiota studies is poorly understood. We investigated the effects of experimental housing circumstances on intestinal microbiota composition in broiler chickens, and how these effects may influence the capacity to determine diet related effects in a nutrition experiment. A cross-sectional experiment was conducted simultaneously in a feed research facility with mesh panels between pens (Housing condition 1, H1), in an extensively cleaned stable with floor pens with solid wooden panels (H2), and in isolators (H3). In H1 and H2 different distances between pens were created to assess gut microbiota exchange between pens. Feed with and without a blend of medium-chain fatty acids (MCFA) was used to create differences in cecal microbiota between pens or isolators within the same housing condition. Male one-day-old Ross broiler chickens (*n* = 370) were randomly distributed across H1, H2, and H3. After 35 days cecal microbiota composition was assessed by 16S ribosomal RNA gene amplicon sequencing. Metabolic functioning of cecal content was assessed based on high-performance liquid chromatography.

**Results:**

Microbial alpha diversity was not affected in broilers fed +MCFA in H1 but was increased in H2 and H3. Based on weighted UniFrac distances, the nutritional intervention explained 10%, whereas housing condition explained 28% of cecal microbiota variation between all broilers. The effect size of the nutritional intervention varied within housing conditions between 11, 27, and 13% for H1, H2, and H3. Furthermore, performance and metabolic output were significantly different between housing conditions. The distance between pens within H1 and H2 did not influence the percentage of shared genera or operational taxonomic units (OTUs).

**Conclusions:**

The cecal microbiota of broilers was modifiable by a nutritional intervention, but the housing condition affected microbiota composition and functionality stronger than the diet intervention. Consequently, for interpretation of intestinal microbiota studies in poultry it is essential to be aware of the potentially large impact of housing conditions on the obtained results.

**Electronic supplementary material:**

The online version of this article (10.1186/s42523-019-0009-z) contains supplementary material, which is available to authorized users.

## Background

Knowledge of the factors that affect the diversity and functioning of intestinal microbes is essential to facilitate the development of new strategies to improve health, to reduce the use of antibiotics and to improve production performance of broiler chickens [1, 2]. Numerous interactions between microorganisms, diet, host and environmental factors affect the composition of the chicken intestinal microbiota [3–5]. Knowledge about how those factors and their interactions shape the intestinal microbiota in broilers is limited but is important for the design and interpretation of experiments, especially for nutritional research. One of those poorly understood factors is the transmission of bacteria present in the living environment to the hosts (environment-to-host transmission) and exchange of microbiota between hosts sharing the same environment (host-to-host transmission), and how this shapes the intestinal microbial communities within hosts [6].

Previous studies have indicated that housing conditions have a major effect on the health of a host and the composition of its intestinal microbiota [7–10]. Broilers raised in isolators showed alteration of the intestinal morphology, with shorter villi, shallower crypts and reduced production of acidic mucin, compared to conventionally raised broilers [11]. This alteration of the intestinal morphology was suggested to be instigated by differences in the bacterial colonization [11]. Piglets raised in an isolator had a different succession of species during the development of the intestinal microbiota than piglets raised under conventional circumstances [12, 13]. The alteration in the intestinal microbiota was associated with an altered expression of immune-related genes [13]. The intestinal microbiota is not only altered in extreme environments, such as isolators, but also in other experimental environments differences in cecal microbiota in broiler chickens were observed [14, 15]. For example, in an experiment with broiler chickens it was observed that both feed intervention as well as housing conditions (i.e. two different experimental rooms that were presumed identical) affected cecal microbiota, with OTUs associated with room being on average approximately 3-fold less predominant than those associated with diet [15].

Another factor related to housing conditions that may shape the intestinal microbiota community is the transmission of microbes between hosts [6]. It is roughly estimated that 50 to 60% of the bacterial genera from the intestinal microbiota of healthy humans produce resilient spores, which are specialized for host-to-host transmission [16]. In humans, it was found that the intestinal microbiota of individuals who live together show less variation between individuals compared to the variation in a group of randomly selected individuals [17, 18]. This has been observed in chickens as well, as the variation between birds within the same pen tended to be smaller than between birds within the same diet group [19, 20]. A study on *Campylobacter jejuni* and *Escherichia coli* showed that spatial distance between pens delayed its transmission from infected to naïve chickens [21]. Consequently, transmission of microbes between spatially separated chickens within a research environment might be an unknown confounding factor. Hence, it is difficult to determine the potential effects of these processes on the reproducibility and outcomes of broiler nutritional interventions. There is a lack of knowledge on the sizes and mechanisms of effects of this transmission of microbes and the exposure to microbes from the environment on broiler intestinal microbiota composition and functioning.

Therefore, the aim of this study was to compare the effect of different experimental housing conditions for broiler chickens on cecal microbiota composition and the concomitant interpretation of a nutritional intervention. The same nutritional intervention was performed simultaneously in three different housing conditions; housing condition 1 (H1), a standard grow-out feed trial facility; housing condition 2 (H2), a facility with floor pens for small-scale experiments; and housing condition 3 (H3), isolators. In H1 and in H2 different distances between pens were created, to observe if distance between pens could influence the intestinal microbiota. Previous studies have shown that the addition of medium-chain fatty acids (MCFA) to feed can significantly change intestinal microbiota composition [22, 23]. Therefore, a diet with and without MCFA was used as a tool to generate differences in cecal microbiota composition between the chickens in different pens within a housing condition. At 35 days of age, we determined cecal microbiota composition based on 16S ribosomal RNA (rRNA) gene amplicon sequence analysis. The metabolic output of the microbes was determined by measuring the production of acetate, butyrate, isobutyrate, lactate, and propionate, and effects on production performance were determined based on body weight on day 35. This research provides insights into the potential effects of interactions between hosts, and hosts and their environment, on the composition and functioning of intestinal microbiota in broiler chickens and the interpretation of nutritional interventions.

## Results

### Biosecurity level among three housing systems

The bacterial loads, as determined using Rodac plates, were different between the housing conditions before the broilers arrived in the experimental facilities (Additional file 1: Figure S1). CFU per Rodac plate were highest in H1 and lower in H2 and H3 (H1-H2, F = 12.1*, p <* 0.001 and H1-H3, F = 10, *p <* 0.001). Based on the Rodac plate results, three out of ten isolators were disinfected again with vaporized hydrogen peroxide before the broilers arrived.

### Dietary effect of MCFA on cecal microbiota in different housing systems

**Fig. 1:**
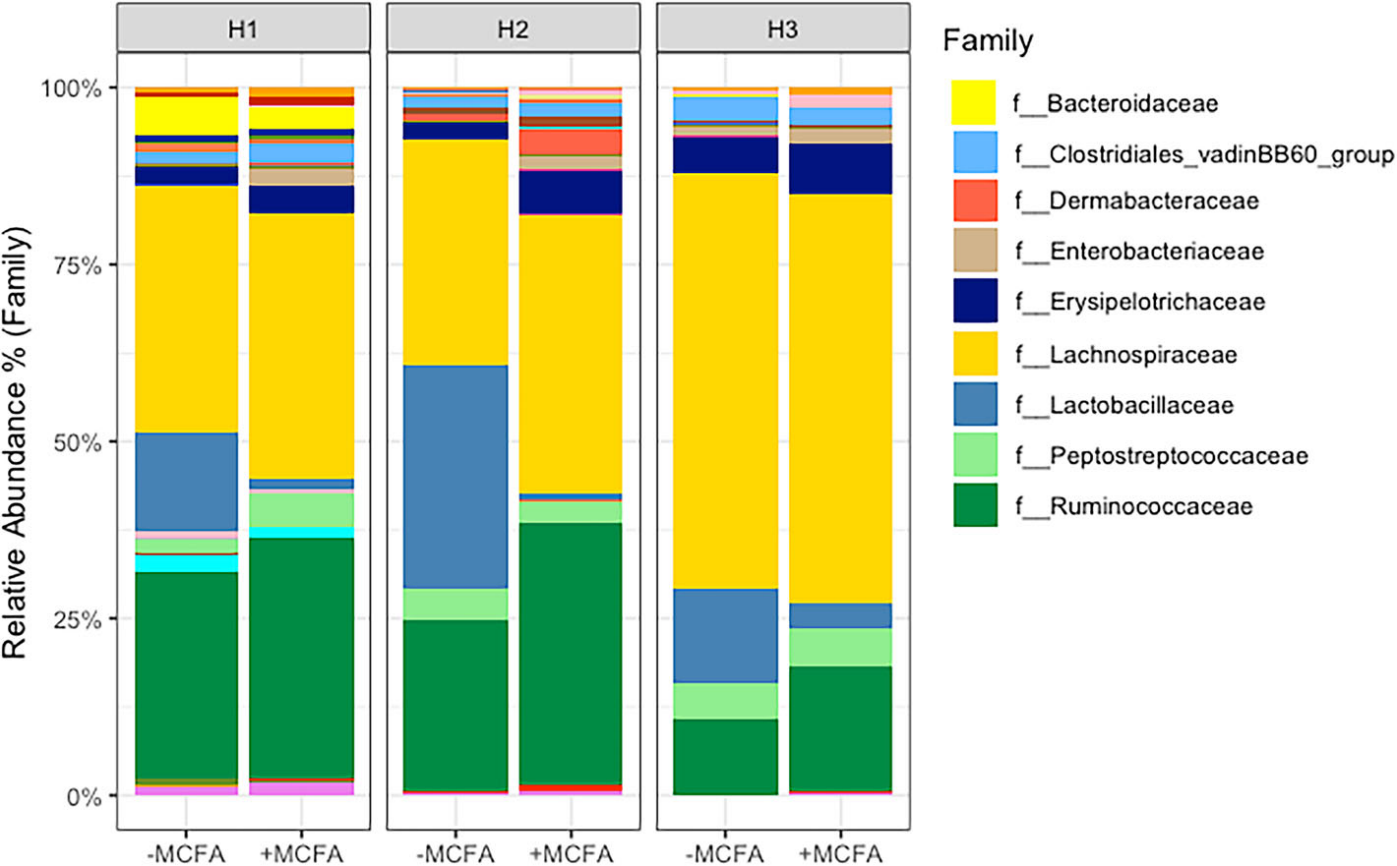
Bar chart with the cumulative relative abundance (%) the legend only contains the most abundant bacterial families. Per housing condition and diet intervention, with an average of 35 birds per bar. For a complete overview of the relative abundance of all 46 families, see Additional file 1: Table S1

On day 35 the cecal content of 210 male Ross 308 broiler chickens from the three different housing conditions were analyzed, with a total of seven cecal samples (i.e. broilers) per pen. Figure 1 provides an overview of the nine most abundant microbial families in the cecal microbiota of the broilers across the three housing conditions and for –MCFA and + MCFA feed (for a complete overview of the relative abundance of all families, see Additional file 1: Table S1).

**Fig. 2:**
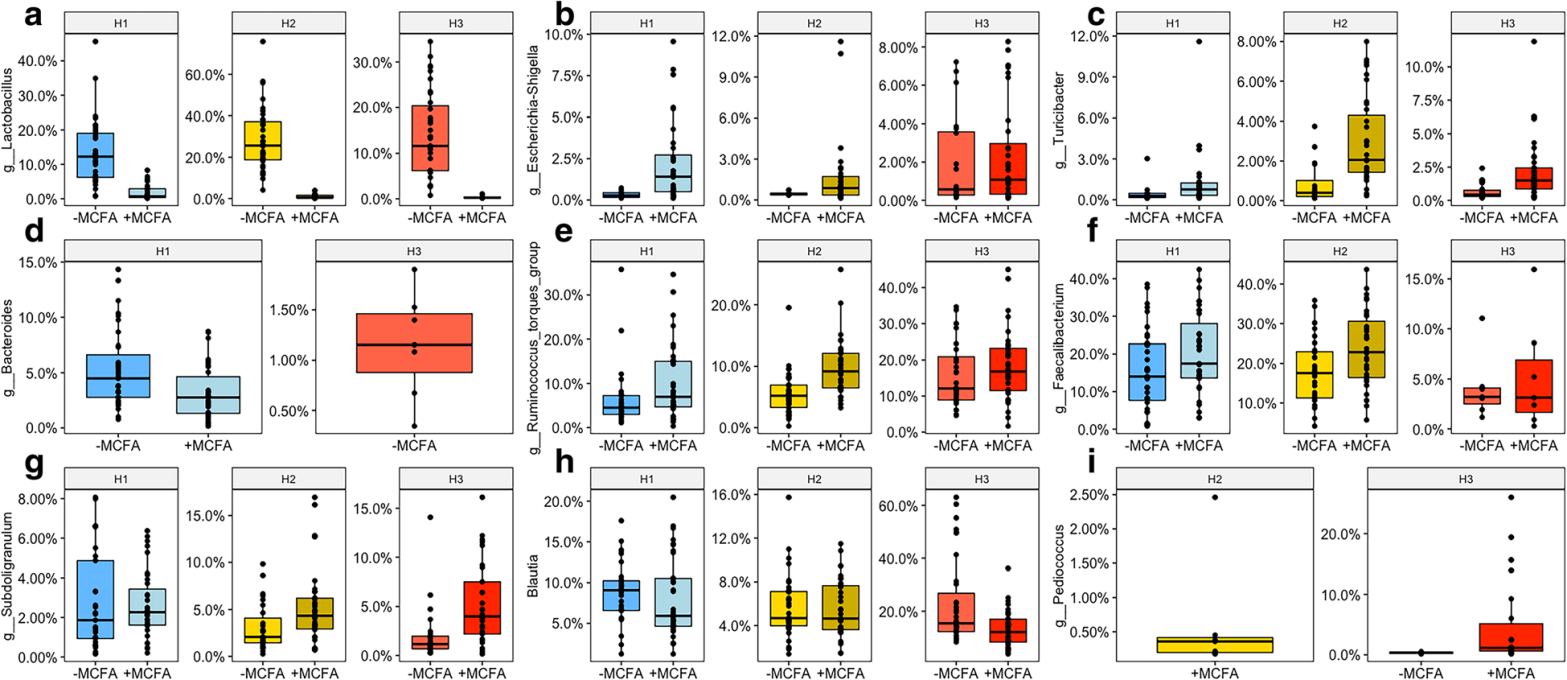
Box plots of nine genera that were significantly different in relative abundance between the broilers on the diet –MCFA or + MCFA. The results are based on differences of relative abundance (Wilcoxon rank-sum test, adjusted *p-*values are corrected *p*-values for multiple testing, BH, < 0.05). The genera *Lactobacillus*, *Escherichia-Shigella* and *Turicibacter* show the same trend across housing conditions (**a-c**), while some effects are unique in a subset of one housing condition (**d-i**)

In the ceca of broilers fed +MCFA, the relative abundance of *Lactobacillus* was significantly lower in all three housing conditions compared to broilers fed –MCFA (Fig. 2a). The reduction in relative abundance for the +MCFA broilers of *Lactobacillus* varied per housing condition, and was 12, 28 and 14% points in H1, H2 and H3 (see Additional file 1: Table S2 for *p*-values). There was a concomitant increase in the relative abundance of *Escherichia-Shigella* and *Turicibacter* in +MCFA broilers in all three housing conditions (Fig. 2b, c).

In addition to the differences that were observed in all housing conditions, some differences were only found in certain housing environments. In H1 the relative abundances of an unknown member of the *Peptostreptococcaceae* family, and the genus *Bacteroides* were lower in +MCFA boilers (Fig. 2d). In H2 the genera *Ruminococcus torques* group*, Fusicatenibacter* and *Subdoligranulum* were higher in +MCFA boilers (Fig. 2e-g). In H3 the relative abundances of the genus *Blautia* (Fig. 2h) and *Clostridium innocuum* group were lower, whereas the relative abundance of an uncultured group within the *Lachnospiraceae* and the genera *Subdoligranulum, Pediococcus* (Fig. 2g, i) and *Erysipelatoclostridium* were higher in broilers fed +MCFA. In total 46 genera differed (adjusted *p* < 0.05) in relative abundance between the feed interventions within one housing condition (Additional file 1: Table S2 and Figure S2).

**Fig. 3:**
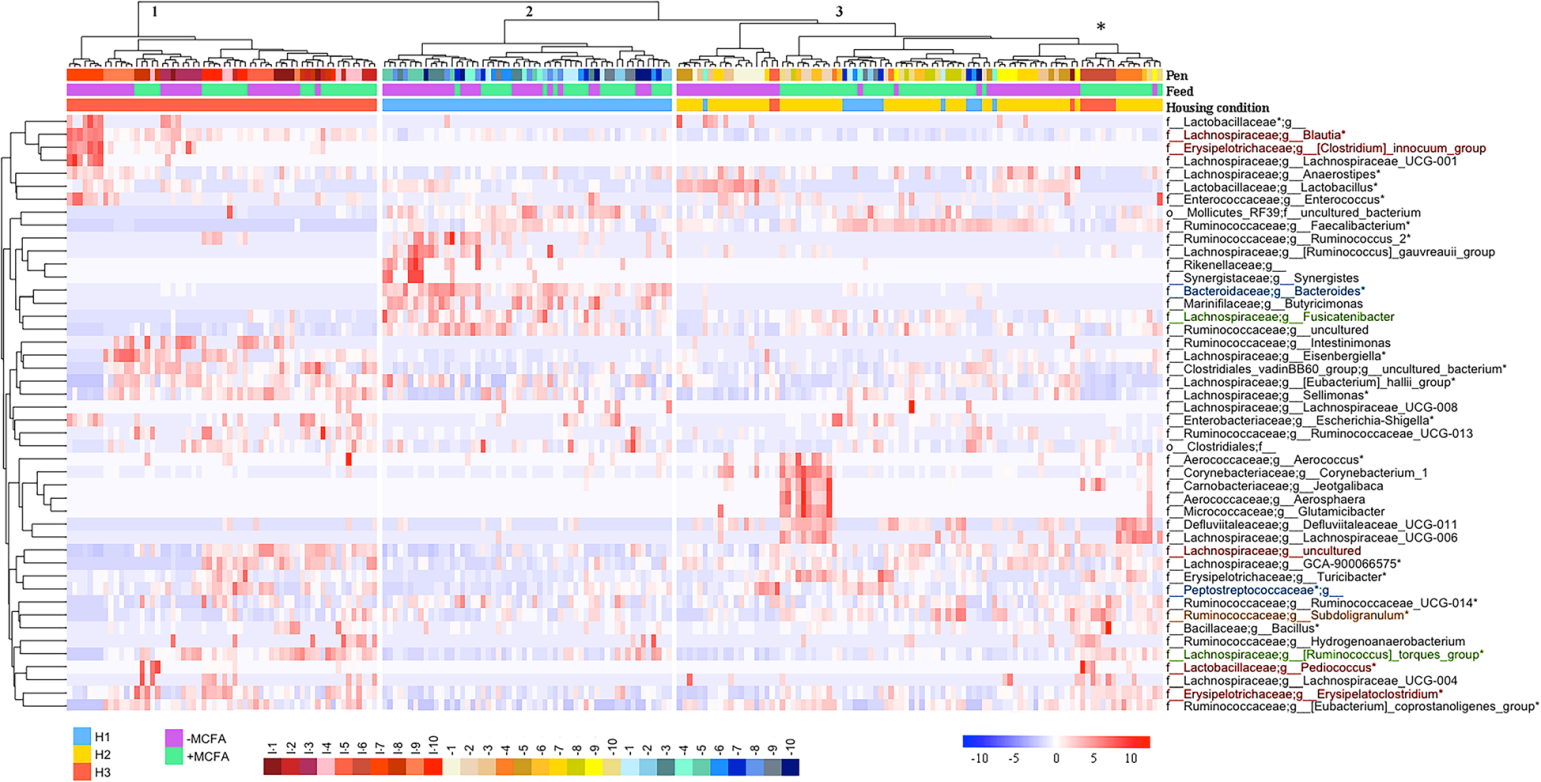
Heatmap of all individual broiler chickens (*n* = 210). The genera that are significant different between the diet intervention within at least one housing condition are shown in this figure (Wilcoxon rank-sum test, adjusted *p*-values are corrected *p*-values for multiple testing, BH, *p* < 0.05). Each red- white -blue dot represents the relative abundance of genera of an individual broiler chicken, of housing condition H1, H2 or H3 (blue, yellow, red) and on diet –MCFA or + MCFA (purple, green). Clustering of broilers is based on Ward’s minimum variance method and based on weighted UniFrac distances matrix. The first three clusters are presented in the figure

The heatmap in Fig. 3 shows all genera that significantly differed in relative abundance between the feed interventions for each housing condition. Hierarchical clustering of broilers revealed three clusters; cluster one coinciding with housing condition H3, cluster two containing most broilers of H1, and the third cluster contained all broilers of H2 and 10 broilers in H3 and 14 broilers in H1 (Fig. 3). Pen effects can be identified, for example, the first seven birds in the first cluster, with a higher relative abundance of *Blautia,* were all raised in the same isolator in H3. Cluster three also contained one isolator of H3 (see *), while in this cluster also eight broilers of H1 cluster together, although these were housed in different pens (Fig. 3). All aforementioned results show that the housing conditions had a larger effect on the microbiota composition than the MCFA feed intervention.

For both –MCFA and + MCFA broilers together, significant differences in relative abundance of genera were also found between housing conditions (Additional file 1: Table S2). In H1 a higher relative abundance of the genus *Bacteriodes* were found compared to H2 (Fig. 2d). In H1 and H2 we also observed a higher relative abundance of *Faecalibacterium* than in H3, and a lower relative abundance for the *Ruminococcus torques* group and *Blautia* (Fig. 2f, e, h). Overall, a large number (*n* = 103) of differences between the housing conditions were observed (total overview in Additional file 1: Table S2 and Figure S2).

### Housing and dietary effect on microbial alpha diversity

**Fig. 4:**
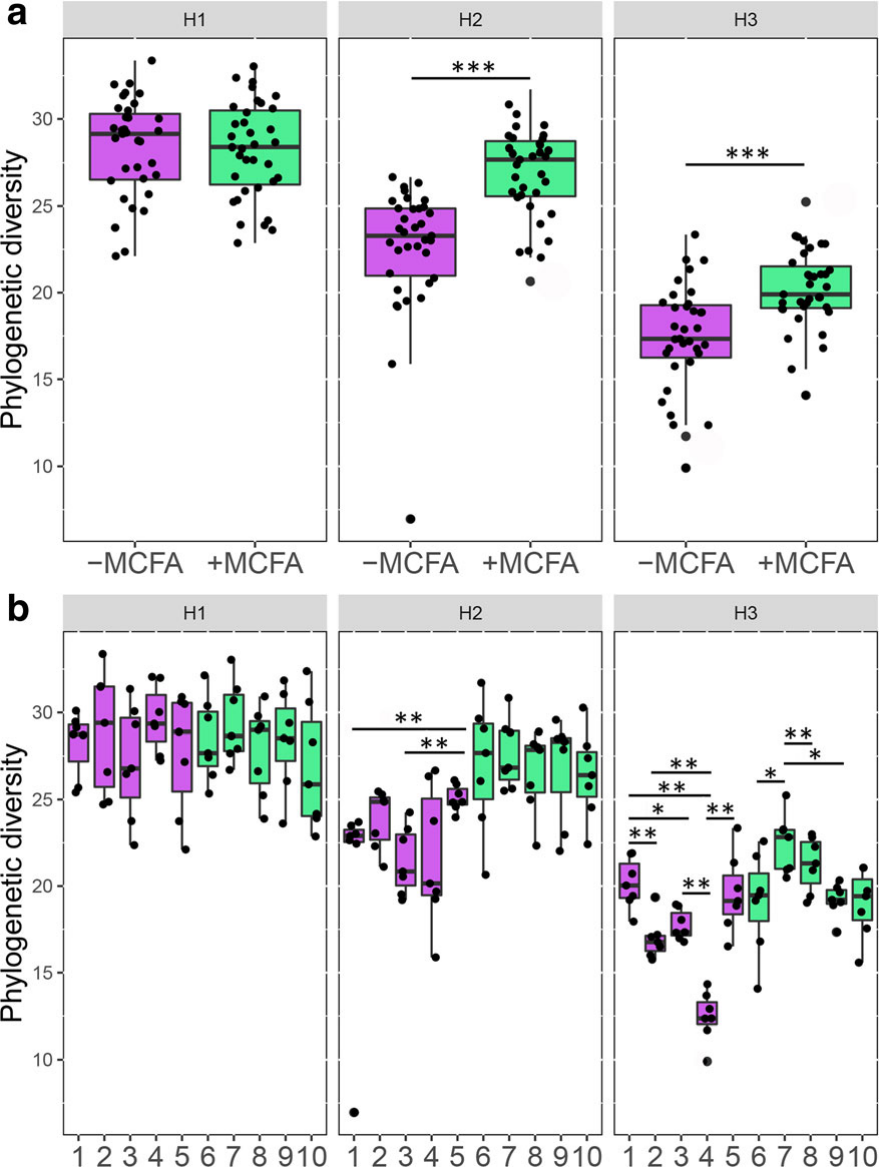
Phylogenetic diversity of the cecal microbiota of the six different experimental groups. **a** The phylogenetic diversity (OTU level) was higher in the ceca of broilers fed +MCFA in H2 and H3 but not in H1. The phylogenetic diversity was highest in H1 and lowest in H3. (*n* = 35 broilers per group, Kruskal-Wallis, * = *p* < 0.05–0.01, ** = *p* < 0.01–0.001, *** = *p* < 0.001). **b** Phylogenetic diversity was different between a subset of pens within the same diet intervention (*n* = 7 broilers per pen)

When comparing cecal microbiota alpha diversity of broilers within the same housing condition, different effects were observed in response to the MCFA intervention. In H1 no effect was observed, whereas in H2 and in H3 the +MCFA resulted in a higher phylogenetic diversity (Fig. 4a). Overall, the phylogenetic diversity was highest in broilers housed in the feed trial facility (H1) and lowest in the isolators, H3 (Fig. 4a). Other alpha diversity metrics were in agreement (Additional file 1: Figure S3). The effect size of the +MCFA was highest in H2 (H1, *X*^*2*^ = 0.03, *p* = 0.846; H2, *X*^*2*^ = 29, *p* < 0.001; H3, *X*^*2*^ = 11, *p* < 0.001). In addition, in housing conditions H2 and H3, but not H1, diversity was different between a subset of the pens within the same intervention (Fig. 4b, Additional file 1: Table S3).

### Housing and dietary effect on microbial beta diversity

**Fig. 5:**
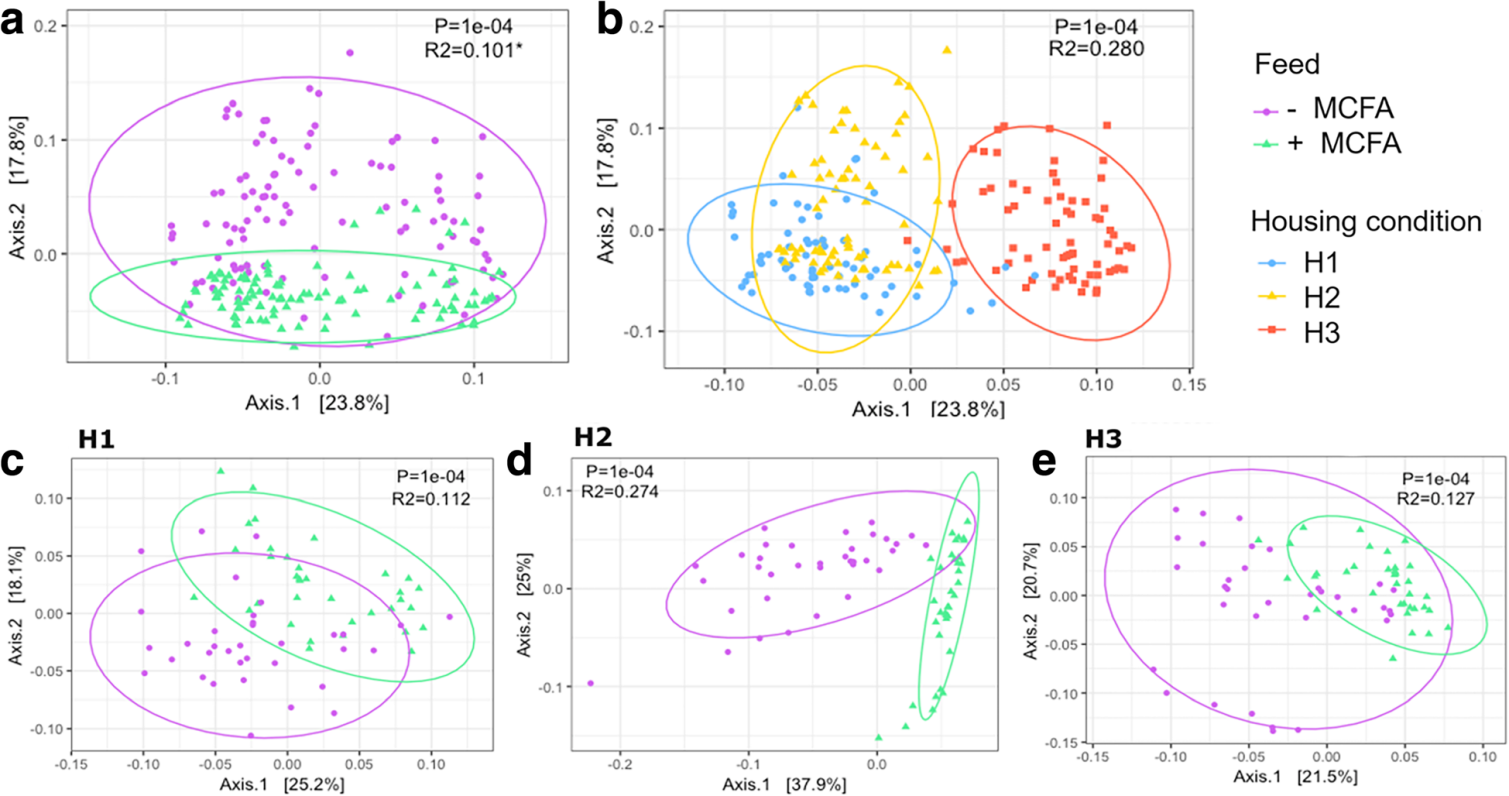
Weighted UniFrac based PCoA analysis across the six different experimental groups. **a** Diet effect across all three housing units (*n* = 210) **b** Housing condition effect **c** Diet effect in housing condition 1 (PERMANOVA, (OTU level), Diet:R^2^ = 11%, *p* < 0.001, Pen:R^2^ = 26%, *p* < 0.001, *n* = 70) **d** Diet effect in housing condition 2 (PERMANOVA, Diet:R^2^ = 27%, *p* < 0.001, Pen:R^2^ = 53%, *p* < 0.001, *n* = 70) **e** Diet effect in housing condition 3 (PERMANOVA, Diet:R^2^ = 13%, *p* < 0.001, Pen:R^2^ = 55%, *p* < 0.001, *n* = 70)

Weighted UniFrac based analysis of cecal microbiota showed that the +MCFA feed intervention explained 10% (R^2^) of microbiota variation independent of the housing condition (Fig. 5a, PERMANOVA, pseudo-F = 23, *p* < 0.001). It should be noted that also the beta dispersion was significantly higher in the –MCFA groups (Fig. 5a; *p* = 0.021). In the total dataset the housing condition explained 28% of microbiota variation (Fig. 5b, pseudo-F = 40, *p* < 0.001, beta dispersion *p* = 0.295).

In H1, diet explained 11% of the total microbiota variation (Fig. 5c, pseudo-F = 9, *p* < 0.001, beta dispersion *p* = 0.968), while in H2, it explained 27% (Fig. 5d, pseudo-F = 26, *p* < 0.001, beta dispersion *p* = 0.450). In H3 the feed intervention explained 13% but beta dispersion was also significantly different between feed groups (Fig. 5e, pseudo-F = 9, *p* < 0.001, beta dispersion *p* = 0.007). Pen explained 26, 53 and 55% of microbiota variation in housing unit H1, H2 and H3. Including pen in the analysis increased the explained variation by definition because we cannot disentangle pen and feed as in each pen broilers were exposed to the same feed intervention. Nevertheless, this suggests the strongest pen effect in H3, since in H3 an increase from 13% (feed only) to 55% (feed and pen) explained microbiota variation was observed.

The above described results were confirmed with Bray-Curtis and Jaccard metrics, which showed that the feed explained most of the variation in H2 and the cage explained most of the variation in H3 (Additional file 1: Figure S4). In contrast, unweighted UniFrac, which only considers presence or absence of OTUs showed that feed explained most variation in H3 instead of H2 (Additional file 1: Figure S4). Across housing conditions, the effect sizes based on unweighted UniFrac were slightly higher than for weighted UniFrac, whereas the opposite trend was observed for corresponding Jaccard and Bray-Curtis dissimilarity (Additional file 1: Figure S4). This suggests that the most abundant taxa were more phylogenetically related (i.e. more similar) compared to the low abundant taxa and that not all of those low abundant taxa were shared between the housing conditions.

### Percentage of microbial taxa shared between pens

We used the percentage of shared genera or shared OTUs as a proxy for putative host-to-host and environment-to-host transmission. The total number of genera identified in each housing condition was 125, 98 and 102, and the average percentage of genera shared between pens was 74, 74 and 55% in H1, H2 and H3. In the pens of H1 and H2 the feed intervention had no effect on percentage of shared genera, i.e. 75% versus 73, and 78% versus 74% (+MCFA and –MCFA, H1: *p =* 0.926, H2: *p =* 0.078). However, in H3 there was a significant difference in shared genera between the two feeds where pens fed +MCFA shared 66% and pens –MCFA 49% (*p =* 0.006). The percentage of shared OTUs was lower compared to percentage of genera, but the trend was the same. Broilers with MCFA in their diet shared more OTUs compared to –MCFA fed broilers (Additional file 1: Table S4). Strikingly, physical distance between pens was not correlated with the percentage of shared OTUs or genera (Additional file 1: Figure S5).

### Metabolic output of the cecum in broiler chickens within and between housing conditions

**Fig. 6:**
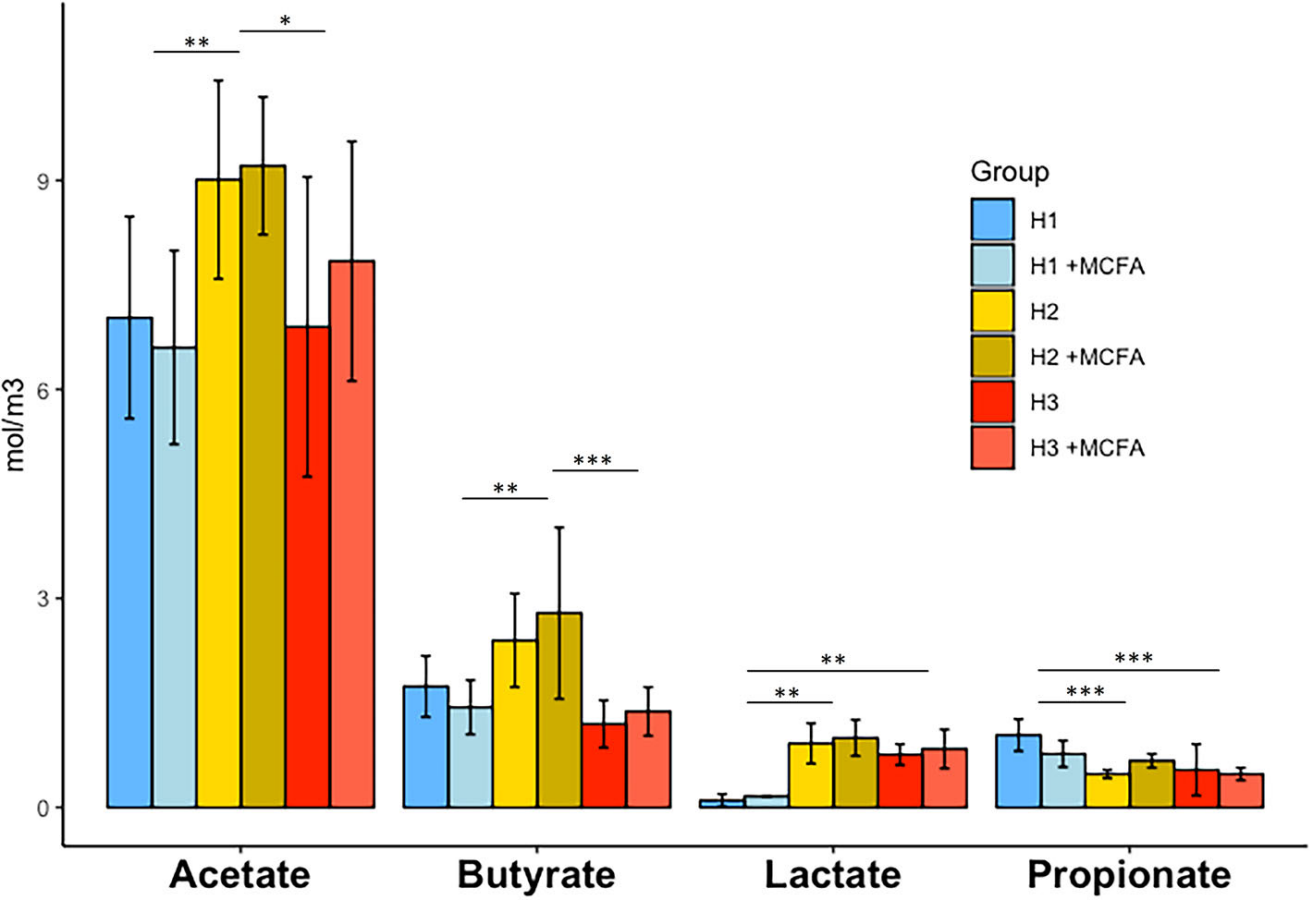
Acetate, butyrate, lactate and propionate concentrations in the cecal content of the six different experimental groups. (*n* = 35 broilers per group, * *p* < 0.05–0.01, ** *p* < 0.01–0.001, *** *p* < 0.001)

Within housing conditions no significant difference between dietary treatment groups was observed for acetate, butyrate, lactate or propionate levels in the cecum (Fig. 6). Isobutyrate was not detected in the cecal samples. However, in contrast, butyrate, acetate, lactate and propionate concentrations were significantly different between housing conditions (Fig. 6), with acetate and butyrate highest in H2 and propionate highest in H1. The concentration of lactate was lowest in H1 (Additional file 1: Table S5).

### Growth performance

**Fig. 7:**
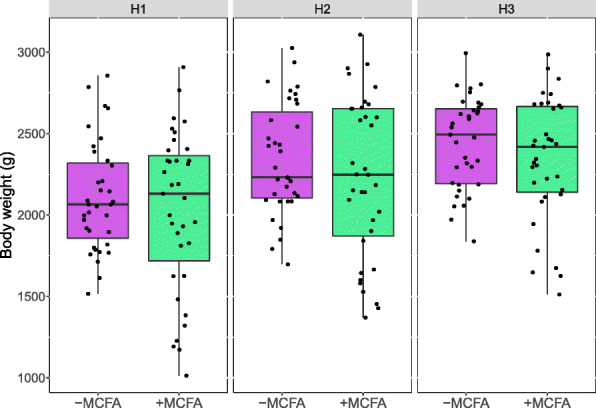
Body weight of broilers on day 35 of age. (*n* = 35 broilers per group)

After 35 days the average body weight was not different between the dietary treatments but differed per housing condition. Body weights of the broilers in H1 were significantly lower than those of broilers in H2 or H3 on the same dietary treatment (Fig. 7, Additional file 1: Table S6). Only in the period 14–35 the average daily gain and the average daily feed intake were lower in +MCFA broilers (Additional file 1: Table S6). In all other measured growth performance data, only housing conditions resulted in different performance (Additional file 1: Table S6).

## Discussion

The aim of this study was to compare the effect of different experimental housing conditions for broiler chickens on cecal microbiota composition and the concomitant interpretation of a nutritional intervention. The variation and composition in the cecal microbiota differed strongly across housing conditions, even with similar management, genetic background, and feed in 35 day old broilers. These findings are in line with previous studies, where housing conditions were shown to influence intestinal microbial composition [7–10, 24]. In addition to the cecal microbiota composition, also metabolic output and growth performance were different between the housing conditions. We also varied the physical distances between pens, to determine the potential effects of exchange of microbes on the outcomes of the experiment. In this study, the distances between pens did not show any significant correlation with the percentages of genera or OTUs shared between pens. However, our results are based on a single observation after 35 days, and hence, we cannot determine whether early in life the distance between pens could have influenced microbiota composition temporarily.

Few studies have been performed on effects of distances between cages on microbial spread [21, 25]. As previously described, small distances could delay the spread of *Campylobacter jejuni* and *Escherichia coli* for days but ultimately did not prevent transmission [21]. How and to what extent an environment influences the composition of the intestinal microbiota, is still unclear, although in humans, it is proposed that spore-forming bacteria influence host-to-host and environment-to-host transmission [6]. Although it is difficult to accurately quantify microbiota transmission, the results of this study strongly suggest differences in the level of transmission of microbes between broilers for the different housing conditions. We found no difference in the alpha diversity between +MCFA and –MCFA fed broilers in H1, while in H2 and H3 the +MCFA broilers displayed a higher cecal microbiota alpha diversity. In addition, the effect of diet or pen on the microbiota composition was lowest in H1. This suggests transmission of microbes may have occurred between the diet groups in H1, and therefore differences between groups due to the diet were leveled out. Additionally, the initial diversity and richness of H1 was higher before the start of the experiment, which might have worked in tandem with the transmission to cause less variation overall in this environment.

In addition to host-to-host transmission, spore-forming bacteria can also influence research environments [15, 16] and this might also be an important factor that influences the intestinal microbiota in broilers. Though, the genera that were either present or absent in the different housing conditions (*Bacteroidetes* and *Pediococcus*), are surprisingly both non-spore-formers. In H3, the isolators, the relative abundances of genera *Faecalibacterium, Blautia,* and *Ruminococcus torques* group were found to be different compared to H1 and H2. These genera are also not known to produce spores. The genus *Bacillus*, known to include endospore-forming bacteria, was present in all housing conditions, while the endospore-forming genus *Clostridium* was only present in H3. Therefore, it is not possible to explain the observed differences between housing conditions by spore-forming bacteria only.

In the H3 there was a low risk of introducing microbes from the shared environment, i.e. surrounding facility or animal technicians, and this might have resulted in a stronger pen effect in H3 compared to H1 and H2. In addition, large differences in exposure to microbes due to differences in levels of biosecurity are likely to have been caused by the different cleaning approaches and downtime between experiments within each housing condition, as suggested by the Rodac plate results. To support this observation, a more extensive characterization of microbial exposure in the different housing conditions should also be included in future studies in order to allow assessing the presence of those microorganisms in the host itself. In one isolator from H3 the relative abundance of the genus *Blautia* was much lower than in the other isolators, and therefore this single isolator had a large impact on the identification of differentially abundant genera independent of the diet intervention. This difference between pens and isolators within the same housing condition might be a result of stochastic variation in the early life microbial colonization of the birds’ intestines [26].

Our observations are in line with a piglet study, that showed that piglets raised in isolators showed a lower microbiota diversity compared to siblings raised at a farm [27]. In addition, also unique genera were observed per housing condition. We succeeded in keeping the temperature and humidity the same across the three housing conditions as well as the light schedule, however, small differences in the local climate of the three units may have occurred. The light intensity in H3 was higher than in H1 and H2. It is not likely that this has had large effects, as light intensity has not been associated with an altered performance [28–30], nor is it known to influence the intestinal microbiota. Despite that we tried to keep all conditions that potentially might influence the intestinal microbiota as consistent as possible, the three housing conditions seem to have their own facility-specific effect on the intestinal microbiota.

Despite the difference in alpha and beta (intra- and inter-individual) diversity across the housing conditions, the +MCFA diet lowered the relative abundance of the genus *Lactobacillus* and heightened the relative abundance of *Escherichia-Shigella* and *Turicibacter* in all three housing conditions*.* This is in line with other studies where MCFA reduced lactobacilli [22, 23]. The observation that *Escherichia-Shigella (*family *Enterobacteriaceae)* was higher in relative abundance in broilers +MCFA for all three housing conditions, is in line with another study that showed that MCFA promoted members of the family *Enterobacteriaceae* in the ileum of broilers [22]. MCFA are also known to control and decrease the spread of pathogens in poultry [31, 32] and improve feed efficiency [22]. However, contrasting effects of *Lactobacillus* spp. on the performance of chickens have been observed earlier [33], possibly because of the different relative abundance of *Lactobacillus* or the presence of different species of *Lactobacillus*. In H1 the genus *Bacteroides* and family *Peptostreptococcaceae* were higher in +MCFA fed broilers, which are both associated with a healthy intestine [6, 34, 35]. In H2 the relative abundances of *Ruminococcus torques* group*, Subdoligranulum,* and *Fusicatenibacter* were heightened in +MCFA fed broilers. Of these, the *Ruminococcus torques* group has been associated with better performance [36]. It has been observed to be more abundant in broilers treated with zinc bacitracin, and those broilers also showed a reduced feed conversion ratio [37]. This correlation of the relative abundance of the *Ruminococcus torques* group with reduced feed conversion ratio was not observed in our data.

The observed lower mean body weight in H1 compared to H2 and H3, may have been caused in part by an infection with the intestinal protozoal parasite *Eimeria* in H1. This is a common infection in commercial broiler chickens, which was prevented by the high biosecurity level and long downtimes between experiments for the other two housing conditions. For instance, *Eimeria* has been described to decrease the richness and diversity of the intestinal microbiota [38], while we found the highest diversity in H1. Also, we found no genera that were previously associated with an *Eimeria* spp. infection [38–40]. Lastly, post mortem and clinical findings suggested that *Eimeria tenella*, the species affecting the ceca, was not present. Thus, a limited effect of the *Eimeria* spp. infection on the microbiota can be expected. In addition, the limited number of birds (*n* = 35) and pens (*n* = 5) to measure differences in performance might also be the reason for not finding clear effects on performance data between +MCFA and -MCFA fed broilers. It is known that substantial variation in performance between individual broilers requires large bird numbers to detect significant differences. For instance, in similar studies 108 or 96 broilers were used to observed potential differences between diet interventions [22, 41].

To characterize the carbohydrate catabolism of the cecal microbiota, lactate and the short chain fatty acids (SCFAs) acetate, butyrate and propionate and were measured. The metabolite analyses supported the observation that the microbiota varied per housing condition. Not only with respect to composition, but also regarding activity. Although, within each housing condition, no difference in metabolic activity was observed while the composition of the microbiota did vary. Thus, similar functions can be exerted by different species and genera. It is therefore important to assess the impact that microbiota has on e.g. intestinal metabolism, rather than only describing which microbial taxa are present.

This study contributes to understanding of the complex underlying mechanisms leading to differences in intestinal microbiota composition and activity in diet intervention experiments and factors confounding these observations. Differences in housing conditions can act as substantial confounding factors in microbiota studies. Although we have not elucidated the exact mechanisms explaining these differences, we have shown that differences with regard to biosecurity level at the start of an experiment, but also with regard to contact with the environment and between pens, may be part of the underlying mechanisms explaining these differences. More knowledge on how to modulate the function of the intestinal microbiota will help to improve the resilience of broiler chickens against pathogens and may reduce the need to use antimicrobial drugs. It is important to realize that in addition to pathogens, also commensals can spread which can impact the reproducibility of microbiota studies [21]. In addition to other known and unknown host- and environmental factors contributing to these observed differences, exposure to microbes present in the living environment is an important factor that can shape the intestinal microbiota community of broiler chickens.

## Conclusion

The same nutritional intervention can modify the intestinal microbiota in the same direction under different housing conditions, however, in this study housing condition affected the microbiota composition and functionality stronger than the nutritional intervention. The unique differences found per housing condition resulted in a different interpretation of the dietary MCFA intervention on the microbial changes. Therefore, it is essential to be aware of the potentially large impact of housing conditions on the interpretation of intestinal microbiota experiments. A challenging task for further nutritional microbiota research is to discover the mechanisms to distinguish transmission between hosts, and between hosts and the experimental environment, to improve the repeatability of microbiota research. To improve understanding of the working mechanisms of diet and the interaction with the intestinal microbiota, nutritional experiments should be repeated and also performed under field conditions, to elucidate the mode of action and access its efficacy.

## Methods

### Experimental design

**Fig. 8:**
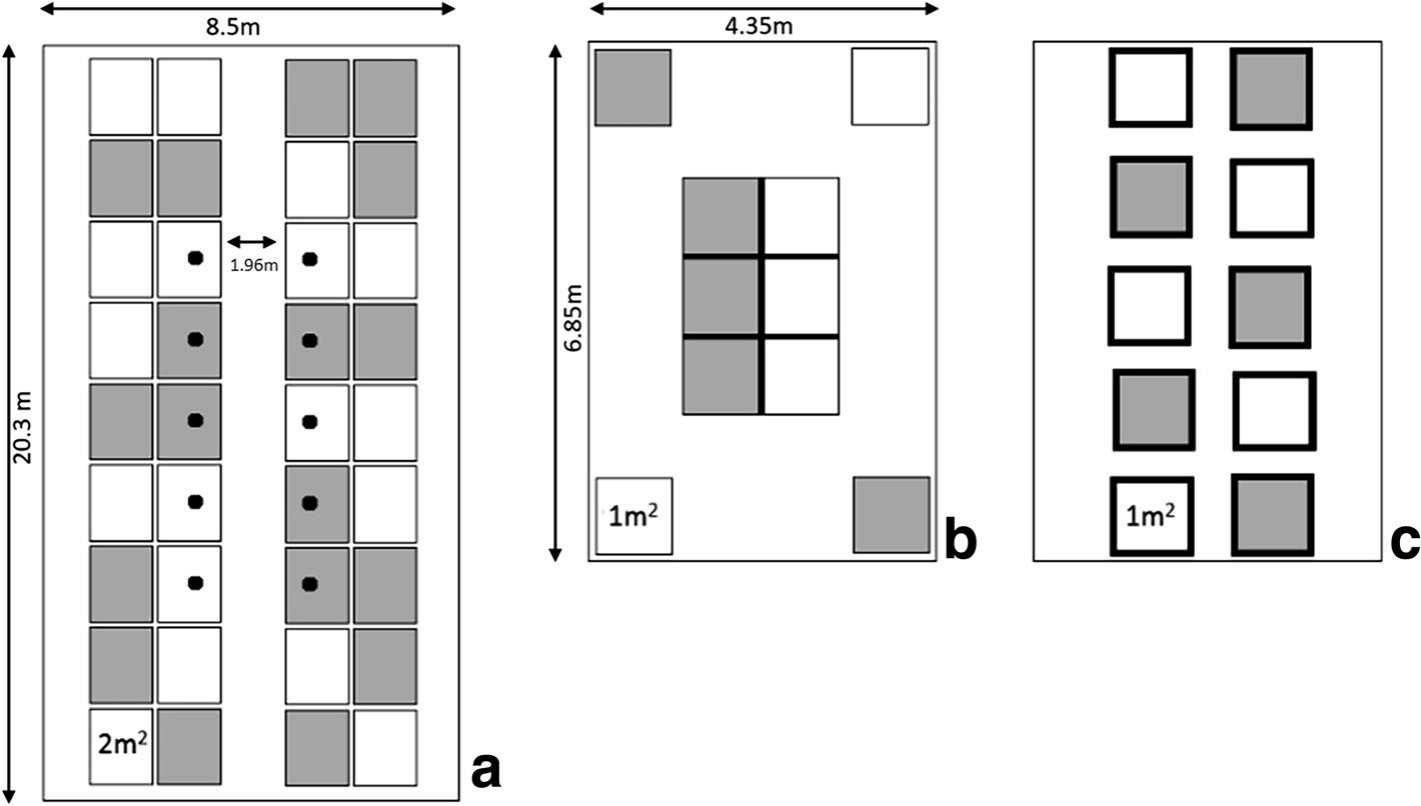
Schematic overview of the three experimental housing conditions. Grey pens are +MCFA, white pens are –MCFA. **a** Housing condition 1 (H1) is a grow-out feed trial facility, with mesh panels separating pens. Only the 10 pens with a dot were individually followed and sampled for 16S rRNA gene amplicon sequencing. **b** Housing condition 2 (H2), an extensively cleaned floor stable with different distances between the pens and adjacent pens separated by solid wooden panels, with only a mesh panel at the front of the pen. **c** Housing condition 3 (H3), isolators, high biosecurity level and protected from environmental contamination

A total of 370 one-day-old male broiler chickens (Ross 308) were purchased from a commercial hatchery (Lagerwey Hatchery, the Netherlands). All chicks were derived from the same 42 week old broiler breeder flock. At the hatchery the chicks were randomly allocated to two different experimental facilities (H1 and H2 + H3). The chicks were transported to these two facilities in the same truck. After a 30 min (H2 & H3) and 50 min’ (H1) drive, the day-old broilers arrived (day 0 of the experiment) and were placed in three different housing conditions (Fig. 8), i.e. H1, a grow-out feed trial facility, H2, a floor stable, and H3, isolators (Additional file 1: Figure S6).

H1 is a research facility at Cargill Animal Nutrition Innovation Center (Velddriel, the Netherlands), and consisted of standard grow-out pens used for broiler feed experiments. A total of 170 chicks were randomly allocated to 10 pens (2.26 m (b) × 0.90 m (w), 2.03 m^2^) (Fig. 8a). The distance between the two blocks separated by an aisle was 1.96 m. In each pen, 10 out of 17 broilers were followed individually throughout the grow-out period. Between the pens steel mesh panels were used as dividers, and the raised metal floor was covered with paper and a 2 cm layer of wood shavings. In this facility a downtime period of two week between experimental rounds was used, and between rounds the facility was cleaned and disinfected with a product with quaternary ammonium compounds and glutaraldehyde (MS Megades, Schippers, the Netherlands).

H2 and H3 were located at the Faculty of Veterinary Medicine of Utrecht University (Utrecht, the Netherlands). Broilers were randomly allocated to H2 or H3. In H2, 100 broilers were randomly distributed over 10 floor pens (1.00 m × 1.00 m, 1 m^2^), in one research unit (Fig. 8b). Adjacent pens were separated by solid wooden panels with only a mesh panel at the front of the pen. A single pen was present in four corners, and in the middle six pens were connected with each other (Fig. 8b). The floor of each pen was covered with a 2 cm layer of wood shavings. Before placement of the chicks, H2 was extensively cleaned and disinfected with vaporized hydrogen peroxide. In H3, 100 broilers were randomly distributed over 10 negative pressure HEPA filtered isolators (0.65 m × 1.5 m, 1 m^2^; Fig. 8c). All materials entering or leaving the isolators were passed through a chlorine tank sealed off with a removable lid. The floor consisted of a box (0.65 m × 0.65 m, 0.42 m^2^) filled with wood shavings to the same amount and from the same batch as in H1 and H2. The other 0.58 m^2^ consisted of a plastic mesh floor. All 10 isolators in H3 were extensively cleaned and disinfected with vaporized hydrogen peroxide before the experiment. There was a downtime period of six weeks with the previous experimental flocks for H2 and H3.

Between H1, H2 and H3 bird management conditions were kept as equal as possible. The wood shavings were transported from H1 to H2 and H3 three weeks before the start of the experiment and were stored under comparable conditions. Although the sizes of the pens were slightly different, the chick densities were the same. After 5 days, the number of chicks was reduced to 15 chicks per pen in H1 and 8 chicks per pen in H2 and H3 this resulted in a stocking density of 7.5 birds per m^2^ for H1, and 8 birds per m^2^ for H2 and H3. In the pens of H1 and H2 artificial lighting was set at 100 lx for 23 h/day (h/d) from day 0–3, 20 h/d from day 4–6 and 18 h/d from day 7–35. In the isolators artificial lighting was set with the same schedule, but with a light intensity of 200–400 lx. Temperature gradually decreased from 34 °C at day 0 with 2.5 °C per week to 20 °C at day 35. Temperature was monitored twice a day and corrected when needed. The birds were observed twice a day, and presence of clinical signs, abnormal behavior and mortality were recorded. At day 7 all birds in all facilities were vaccinated against Newcastle Disease virus (Avinew® Neo, Boehringer Ingelheim, Germany) with the same battery-operated backpack sprayer (H1 and H2) or handheld garden sprayer (H3).

### Experimental feeds

All broilers had ad libitum access to feed and water throughout the experimental period. To establish differences in intestinal microbiota, feeds with and without MCFA (+MCFA and –MCFA) were used [22]. The two different feeds were formulated to meet the nutrient requirements of broilers and based on digestibility and nutrient data provided by the Feed Tables from the Dutch Central Bureau of Livestock Feeding (CVB, 2016). For each feeding phase, a starter and grower basal feeds were produced. Starter and grower feeds contained 2,850 and 2,925 kcal of apparent metabolizable energy (AME)·kg^− 1^ and 10.48 and 9.87 g·kg^− 1^ apparent fecal digestible lysine. The feed was wheat-corn soybean meal based and in the +MCFA feed, a blend of 0.3% C10:0 capric acid and 2.7% C12:0 lauric acid (Sigma-Aldrich, the Netherlands) was added. Diets were kept isocaloric by exchanging the MCFA blend with soybean oil and animal fat based on the ingredient energy values. Diets were produced at Research Diet Services (the Netherlands) and pelleted using steam addition (approximately 80 °C) at 2.5 mm (starter feeds; 0 to 14 days of age) and at 3.0 mm (grower feeds; 14 to 35 days of age). Diets did not contain antimicrobial additives.

### Data collection

After cleaning and disinfection of the three housing units (H1–3), a hygienogram was made with Replicate Organism Detection And Counting (Rodac) plates. The Rodac plates (55 mm diameter) contained medium with 16 g/l agar, 1 ml/l tween 80, 1 g/l ammonium carbonate, 2 g/l lecithin, 1 g/l l-histidine, 5 g/l sodium chloride, 10 g/l meat extract, 10 g/l peptone (tryptone + meat peptone) and 0.5 g/l sodium thiosulphate (5H_2_O) (GD Animal Health, the Netherlands). In every pen at least one Rodac plate was pressed gently on a surface for 30 s. After incubation for 24 h at 38 °C the number of colonies was counted to determine the number of colony forming units (CFU).

In all three housing conditions, individual broiler weights were recorded at the start of the experiment (day 0) and at days 14 and 35. In addition to body weights, also feed consumption was recorded for each pen. The gain to feed ratio was calculated as kg of weight gain/kg of feed consumed for each time period (0 to 14, 14 to 35 and 0 to 35 days of age). On day 35, all broilers in the different housing units were euthanized, using carbon dioxide (H1) or electrocution followed by cervical dislocation (H2 and H3). A pen from H2 and H3 were selected alternately for euthanasia, to avoid a sampling effect due to time differences between housing conditions. Cecal content of each broiler was gently squeezed into sterile cryotubes and snap frozen on dry ice and stored at − 80 °C for microbial genomic DNA extraction. Between sampling of each broiler sterile gloves were changed, and the table, scissors and tweezers were cleaned with 70% ethanol to prevent cross contamination between samples.

### DNA extraction

DNA was extracted, from 0.25 g cecal content, using 700 μl Stool Transport and Recovery (STAR) buffer (Roche Diagnostics Nederland BV, the Netherlands). The cecal sample was transferred to a sterile screw-capped 2 ml tube (BIOplastics BV, the Netherlands) containing 0.5 g of zirconium beads (0.1 mm; BioSpec Products Inc., USA) and 5 glass beads (2.5 mm; BioSpec Products Inc., USA). The samples were treated in a bead beater (Precellys 24, Bertin technologies, France) at a speed of 5.5 ms^− 1^ for 3 × 1 min, followed by incubation at 95 °C with agitation (15 min and 300 rpm). The lysis tube was centrifuged (13,000 g for 5 min at 4 °C), and the supernatant was transferred to a 2 ml microcentrifuge tube. Thereafter, the above described process was repeated with 300 μl of STAR buffer. An aliquot (250 μL) of the combined supernatants from the sample lysis was then transferred into the custom Maxwell® 16 Tissue LEV Total RNA Purification Kit cartridge. The remainder of the extraction protocol was then carried out in the Maxwell® 16 Instrument (Promega, the Netherlands) according to the manufacturer’s instructions. DNA concentration was measured with a NanoDrop ND-1000 spectrophotometer (NanoDrop® Technologies, USA), and DNA was stored at − 20 °C until further use.

### Microbiota composition

Extracted DNA was diluted to 20 ng/μL in nuclease free H_2_O. All PCR plastics were UV irradiated for 15 min before use. For 16S rRNA gene-based microbial composition profiling, barcoded amplicons covering the variable regions V5-V6 of the 16S rRNA gene were generated by PCR using the 784F and 1064R primers [42].

Each sample was amplified in duplicate using Phusion hot start II high fidelity polymerase (Finnzymes, Finland), checked for correct size and concentration on a 1% agarose gel and subsequently combined and purified using CleanNA magnetic beads (CleanNA the Netherlands). The 50 μl PCR reactions contained 36.5 μL nucleotide free water (Promega, USA), 0.4 μL of 2 U/μl polymerase, 8 μL of 5 × HF buffer, 1 μl of 10 μM stock solutions of each of the forward (784F) and reverse (1064R) primers, 1 μL 10 mM dNTPs (Promega) and 1 μL template DNA.

Reactions were held at 98 °C for 30 s and amplification proceeded for 25 cycles at 98 °C for 10 s, 42 °C for 10 s, 72 °C for 10 s and a final extension of 7 min at 72 °C. Synthetic communities of known composition were added as positive controls [42], and samples with nuclease free water were added as no-template negative controls to ensure high quality sequencing data. A composite sample for sequencing was created by combining equimolar amounts of amplicons from the individual samples, followed by a final purification step with magnetic beads to remove any remaining contaminants. The resulting libraries were sent to GATC Biotech (Germany; now part of Eurofins Genomics Germany GmbH) for sequencing on an Illumina Hiseq2500 instrument.

Data was analyzed using NG-Tax [42]. In short, paired-end libraries were filtered to contain only read pairs with a perfect match to the primers and perfectly matching barcodes, to demultiplex reads by sample. OTU were defined as unique sequences. The OTU picking strategy was based on an open reference approach. First, reads were sorted by abundance per sample and OTUs with an abundance of < 0.1% were discarded. In a second step the remaining reads were matched to the first set of OTUs allowing for one mismatch. Taxonomy was assigned using SILVA 128 16S rRNA gene reference database [43].

### High-performance liquid chromatography (HPLC)

After DNA extraction, from the same 2 ml cryo tubes, 100 mg of cecal content was diluted in 900 μl Milli Q, and centrifuged (13,000 g for 15 min at 4 °C). Supernatant was stored at − 20 °C until HPLC analysis. Crotonate was used as internal standard, and the external standards were acetate, butyrate, isobutyrate, lactate and propionate. Substrate conversion and product formation were measured with a Spectrasystem HPLC (Thermo Scientific, the Netherlands) equipped with a Shodex SUGAR SH1011 column with guard column SUGAR KS-G 6B (Agilent, the Netherlands) for the separation of organic acids and carbohydrates. Measurements were conducted at a column temperature of 45 °C with an eluent flow of 0.8 ml min − 1 flow and the detector RID 20a.

### Statistical analysis

All statistical analyses were performed in R version 3.4.2 (R Foundation for Statistical Computing, Austria [44]), using the packages: Phyloseq, Microbiome, and Vegan [45–47]. To test for differences in relative abundance of genera between two groups, we used a Wilcoxon rank-sum test and corrected for multiple testing with Benjamini-Hochberg (BH). Alpha diversity (within sample) was determined using phylogenetic diversity [48], Shannon, Inverse Simpson and Fisher. Faiths phylogenetic diversity not only takes into account the numbers of bacteria, but also the phylogenetic relatedness of those bacteria [48]. Beta diversity (between samples) was determined using Jaccard, Bray-Curtis, weighted and unweighted UniFrac metrics [49–51]. Differences in alpha diversity between treatment groups were tested with a Kruskal-Wallis test and pairwise comparisons were tested using a Wilcoxon rank-sum test. Multivariate microbiota data were visualized using principal coordinates analysis (PCoA), and non-parametric permutational analysis of variance (PERMANOVA) tests were used to analyze group differences within multivariate community data [52]. Growth performance data (body weight, average daily gain, average daily feed intake and gain to feed ratio) and concentrations of butyrate, acetate, propionate and lactate concentrations were analyzed with ANOVA test with Tukey’s post-hoc test, using pen as experimental unit.

## Additional file

Additional file 1: **Figure S1.** Rodac plate results. **Figure S2.** The relative abundance of genera (alphabetic) that were significantly different between broilers on the nutritional intervention within the housing condition or between housing conditions. **Figure S3.** Alpha diversity based on Shannon, Inverse Simpson and Fisher showed the same trend as the phylogenetic diversity. **Figure S4.** Principle Coordinates Analysis based on Bray-Curtis, Jaccard, Unweighted UniFrac and Weighted UniFrac distance matrices. **Figure S5.** Percentage of microbial taxa shared between pens within a housing condition. **Figure S6.** Housing conditions (H1-H3). **Table S1.** Cecal microbiota composition at family level per housing condition and nutritional intervention. **Table S2.** Abundance testing for genera that were significantly different between the chicks on +MCFA or -MCFA feed and between the housing conditions. **Table S3.** Pairwise comparison of phylogenetic diversity within housing conditions and between pens. **Table S4.** Total number of genera or OTUs per pen, and the total number of genera or OTUs shared between pens. **Table S5.** Effect of dietary treatment and housing condition on mean concentrations of acetate, butyrate, propionate and lactate. **Table S6.** Effect of dietary treatment and housing condition on mean body weight, average daily gain, feed intake, and gain to feed ratio. (DOCX 5012 kb)

## Data Availability

Availability of data and materials Raw sequence data were submitted into the Sequence Read Archive (SRA) at the NCBI under accession number PRJNA553870.

## Abbreviations

16S rRNA: 16 Svedberg ribosomal ribonucleic acid
AME: Apparent metabolizable energy
ANOVA: Analysis of variance
C: Celsius
CFU: Colony forming units
g: Gram
h/d: Hours/day
HPLC: High-performance liquid chromatography
lux: Illuminance
MCFA: Medium-chain fatty acids
OTU: Operational taxonomic units
PCoA: Principal coordinate analysis
PCR: Polymerase-chain reaction
PERMANOVA: Permutational multivariate analysis of variance
Rodac: Replicate organism detection and counting
STAR: Stool transport and recovery
UniFrac: Unique fraction
