# Supplementary material for: Take care of the environment: housing conditions affect the interplay of nutritional interventions and intestinal microbiota in broiler chickens
Source: Anim Microbiome. 2019 Aug 27;1:10. doi: 10.1186/s42523-019-0009-z (PMC7807522; doi:10.1186/s42523-019-0009-z)
Supplement: Supplementary file 1 — Figure S1. Rodac plate results. Figure S2. The relative abundance of genera (alphabetic) that were significantly different between broilers on the nutritional intervention within the housing condition or between housing conditions. Figure S3. Alpha diversity based on Shannon, Inverse Simpson and Fisher showed the same trend as the phylogenetic diversity. Figure S4. Principle Coordinates Analysis based on Bray-Curtis, Jaccard, Unweighted UniFrac and Weighted UniFrac distance matrices. Figure S5. Percentage of microbial taxa shared between pens within a housing condition. Figure S6. Housing conditions (H1-H3). Table S1. Cecal microbiota composition at family level per housing condition and nutritional intervention. Table S2. Abundance testing for genera that were significantly different between the chicks on +MCFA or -MCFA feed and between the housing conditions. Table S3. Pairwise comparison of phylogenetic diversity within housing conditions and between pens. Table S4. Total number of genera or OTUs per pen, and the total number of genera or OTUs shared between pens. Table S5. Effect of dietary treatment and housing condition on mean concentrations of acetate, butyrate, propionate and lactate. Table S6. Effect of dietary treatment and housing condition on mean body weight, average daily gain, feed intake, and gain to feed ratio. (DOCX 5012 kb) [file 42523_2019_9_MOESM1_ESM.docx]

# Additional file

Figure S1. **Rodac plate results.**

In H1 a mean of 46.5 (SD = 45.5) CFU were found per Rodac plate). In H2 this was 4.7 (3.7) CFU and in H3 2.7 (19.6) CFU per Rodac plate. ANOVA. H1-H2. F=12.1*. P<0.001* and H1-H3. F=10. *P<0.001*.


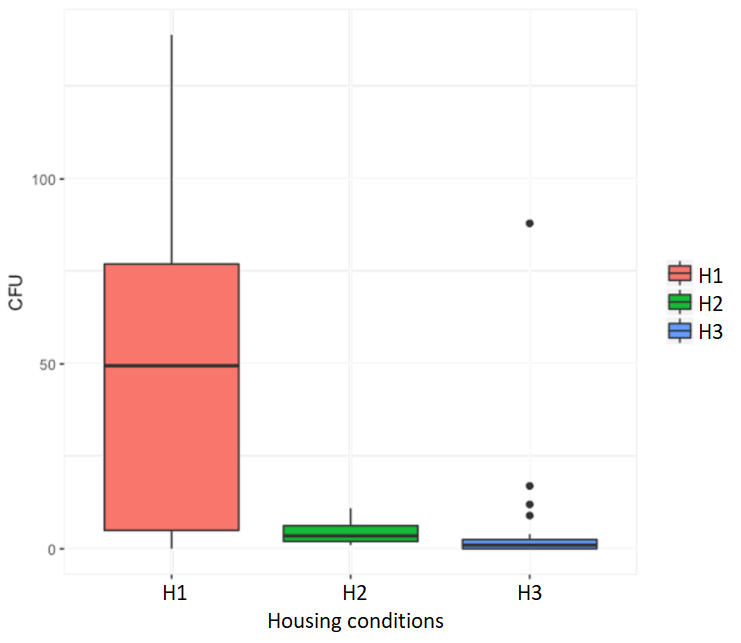


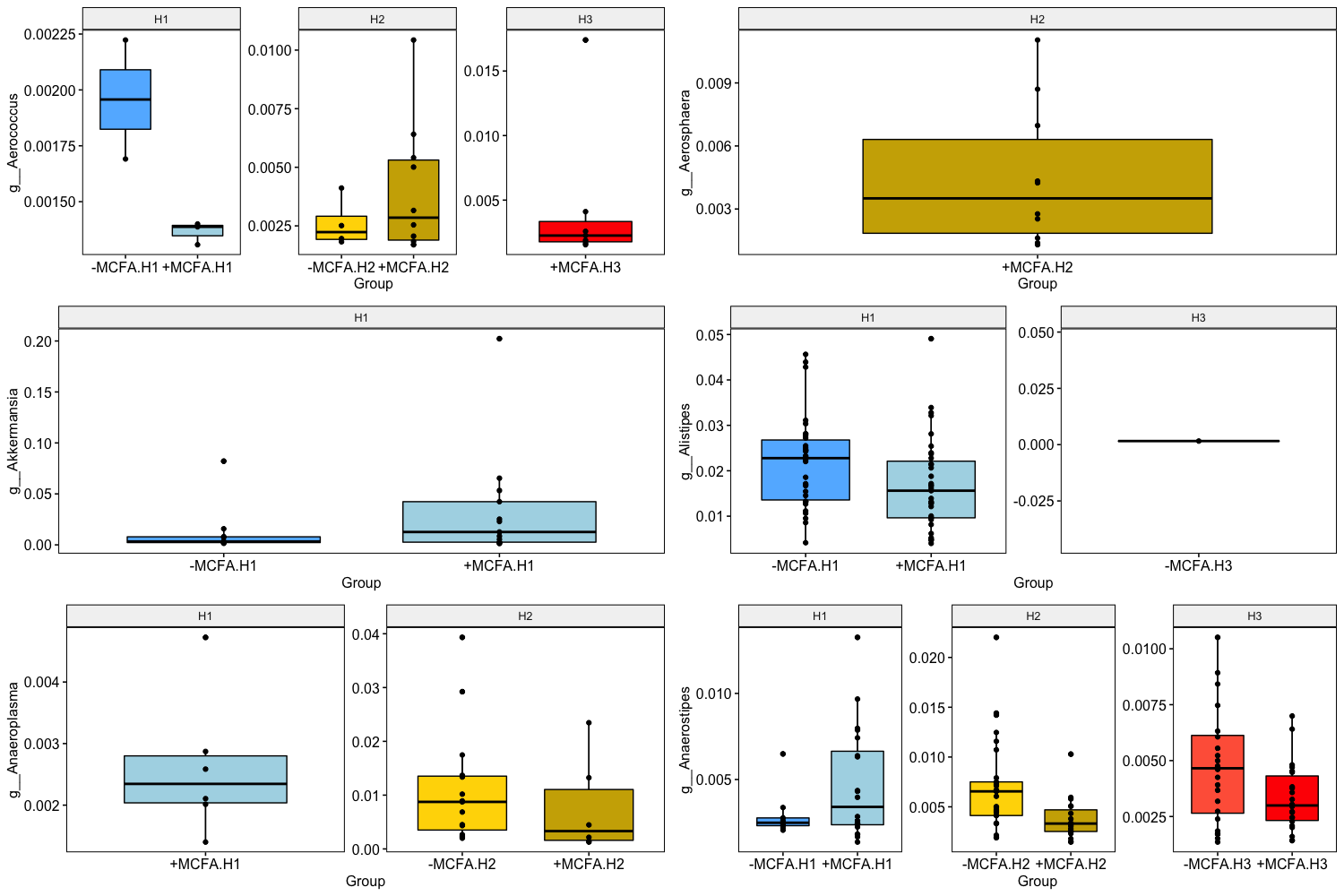
**Figure S2. The relative abundance of genera (alphabetic) that were significantly different** **between broilers on the nutritional intervention within the housing condition or between housing conditions** Each dot represents one sample, data is presented as proportions (0-1)
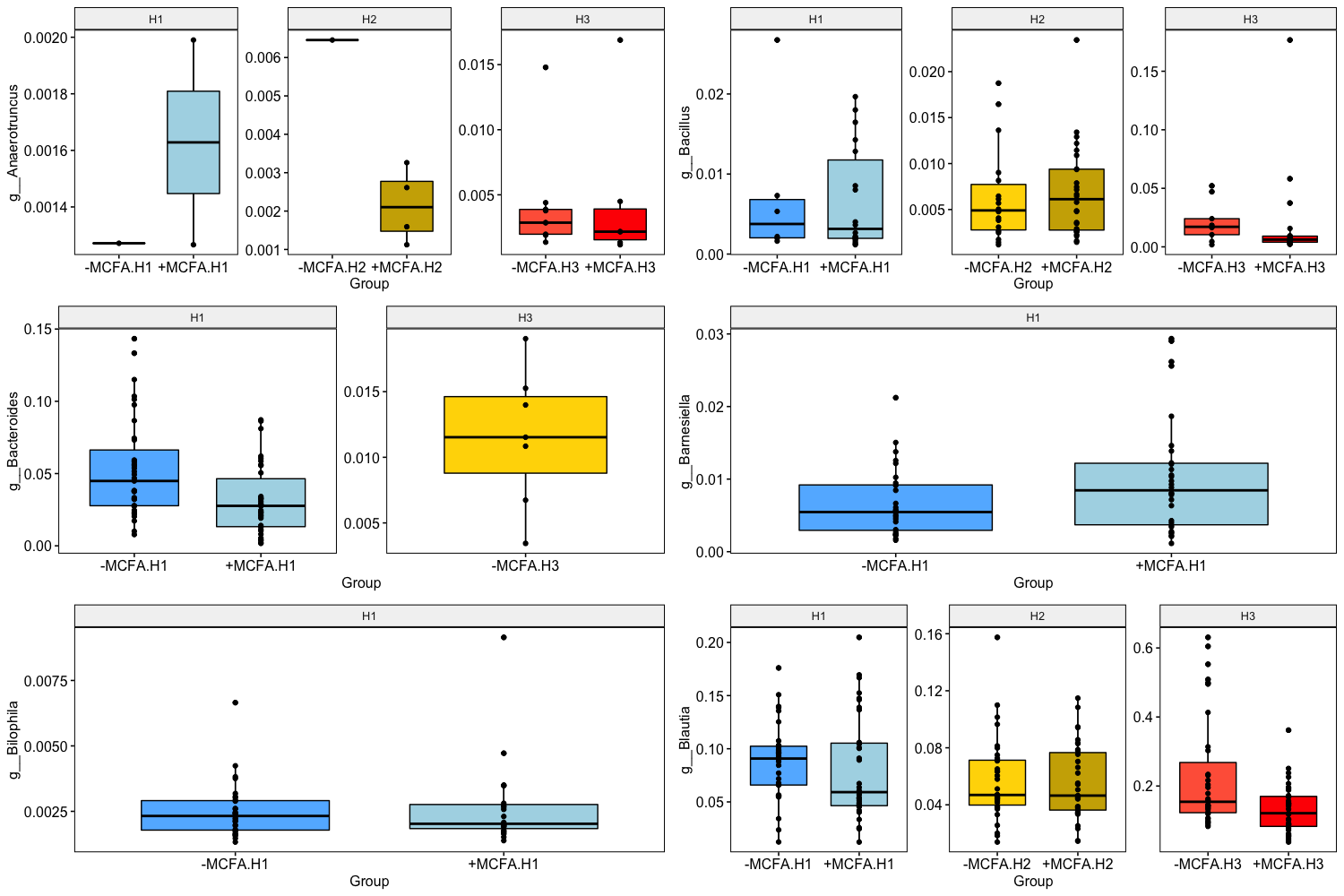
, (Wilcoxon rank-sum test, corrected for multiple testing)


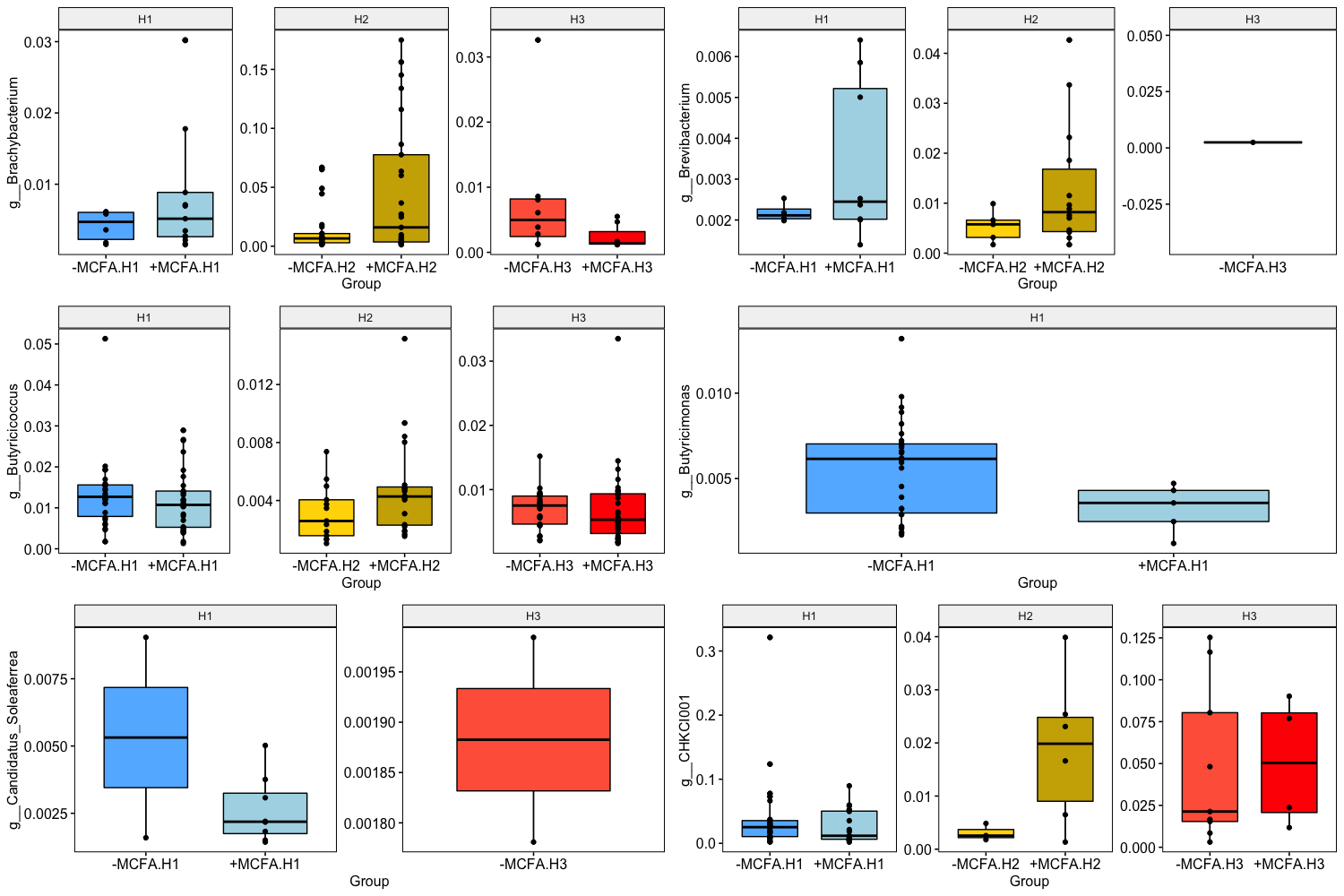

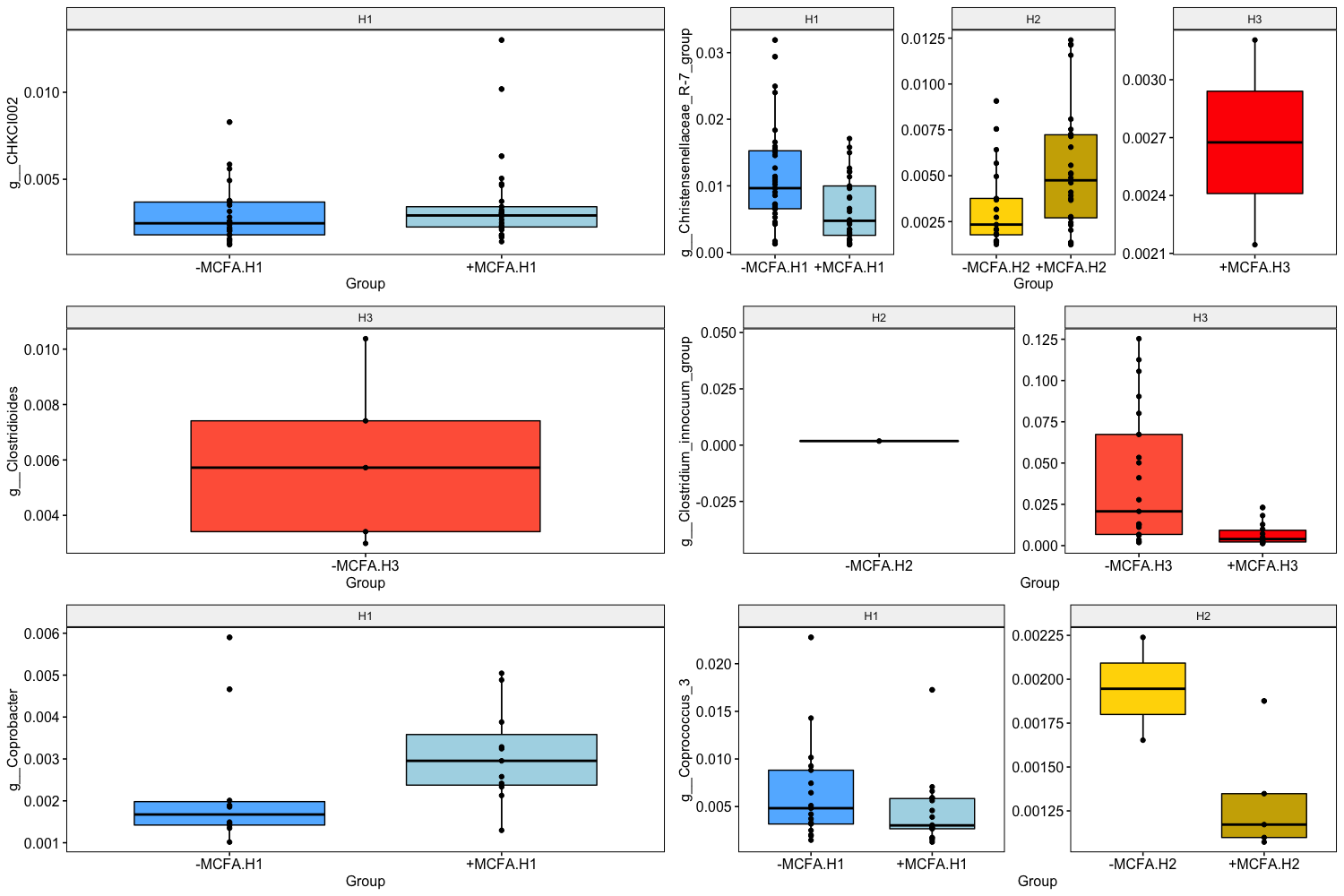


**
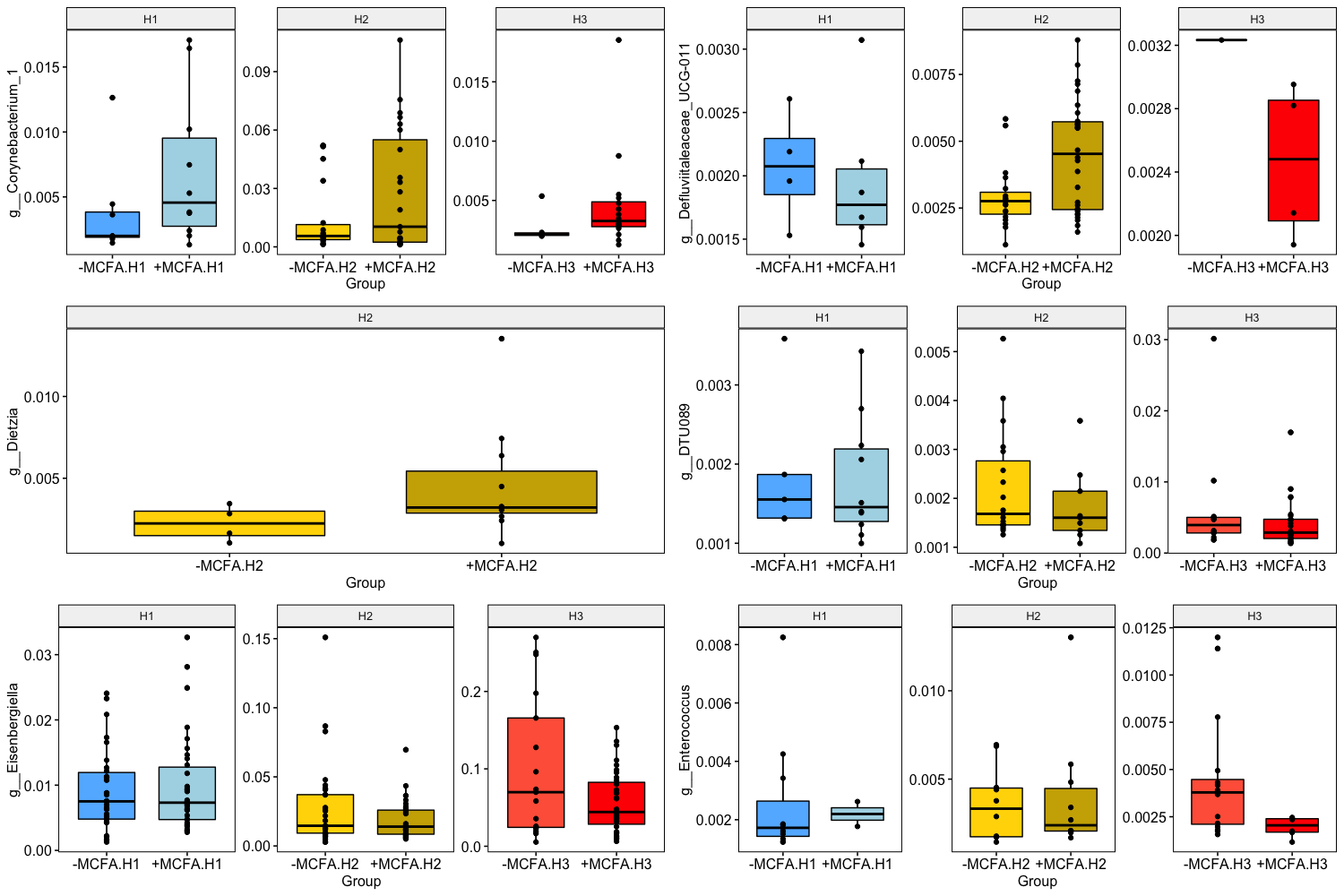

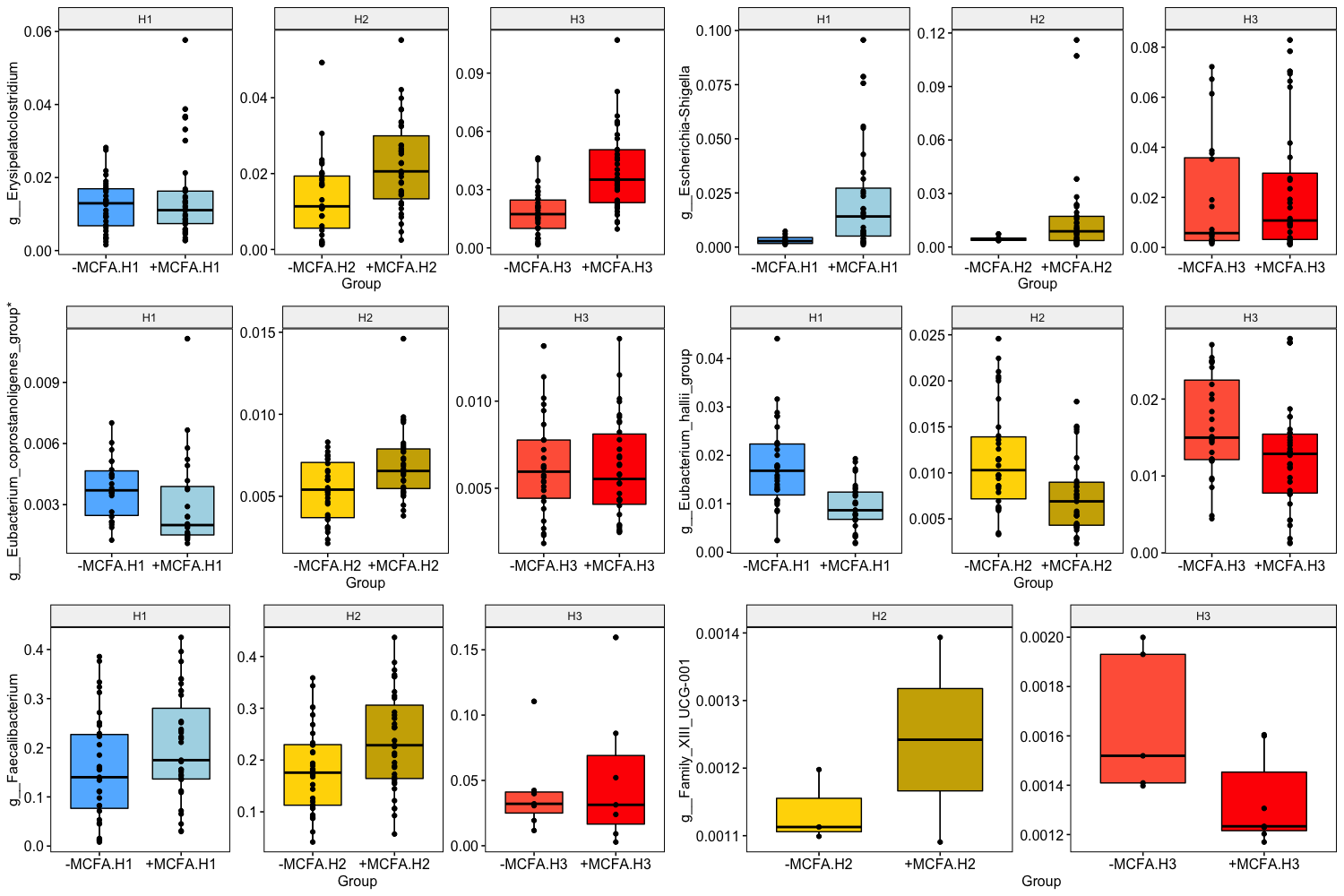
**

**
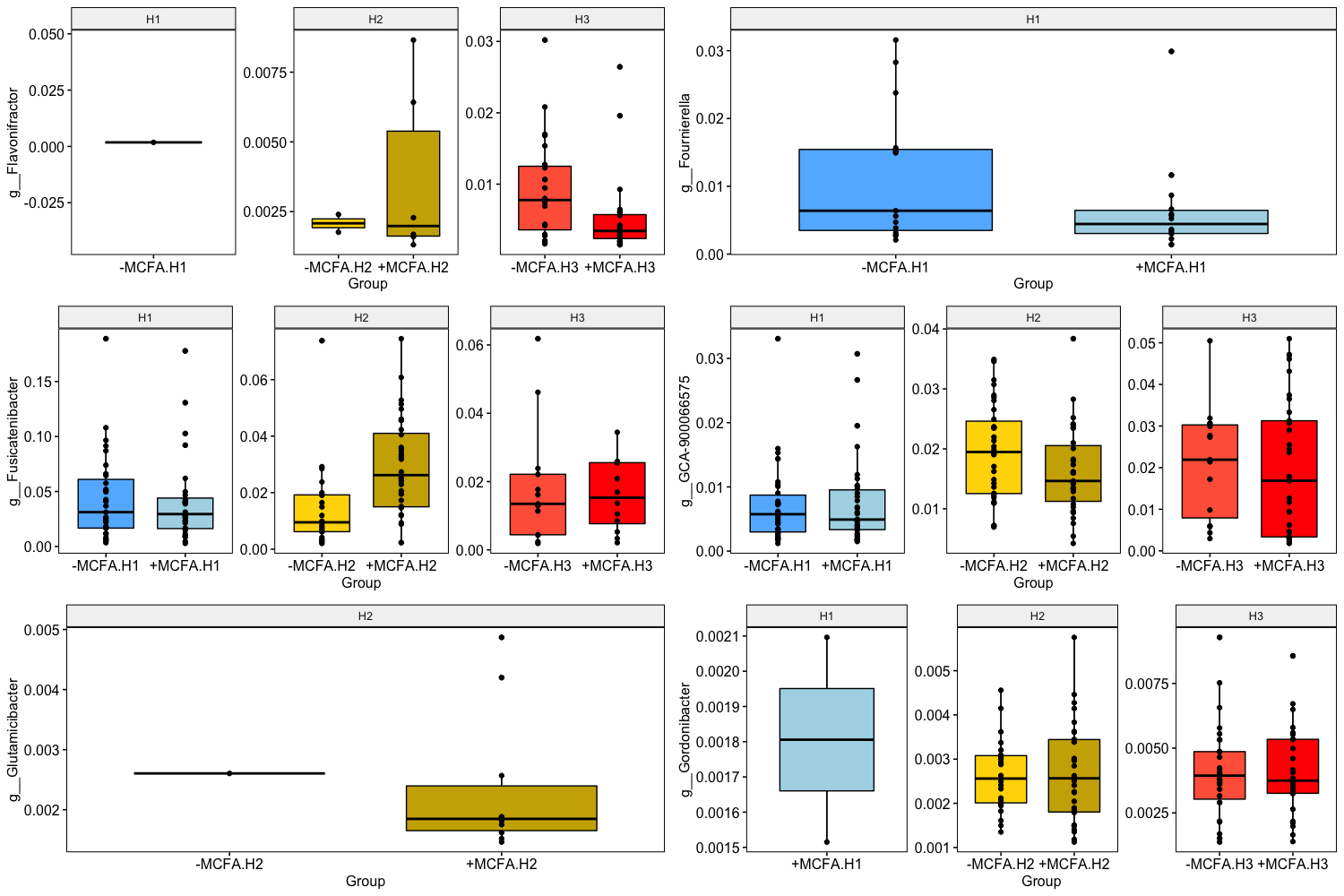

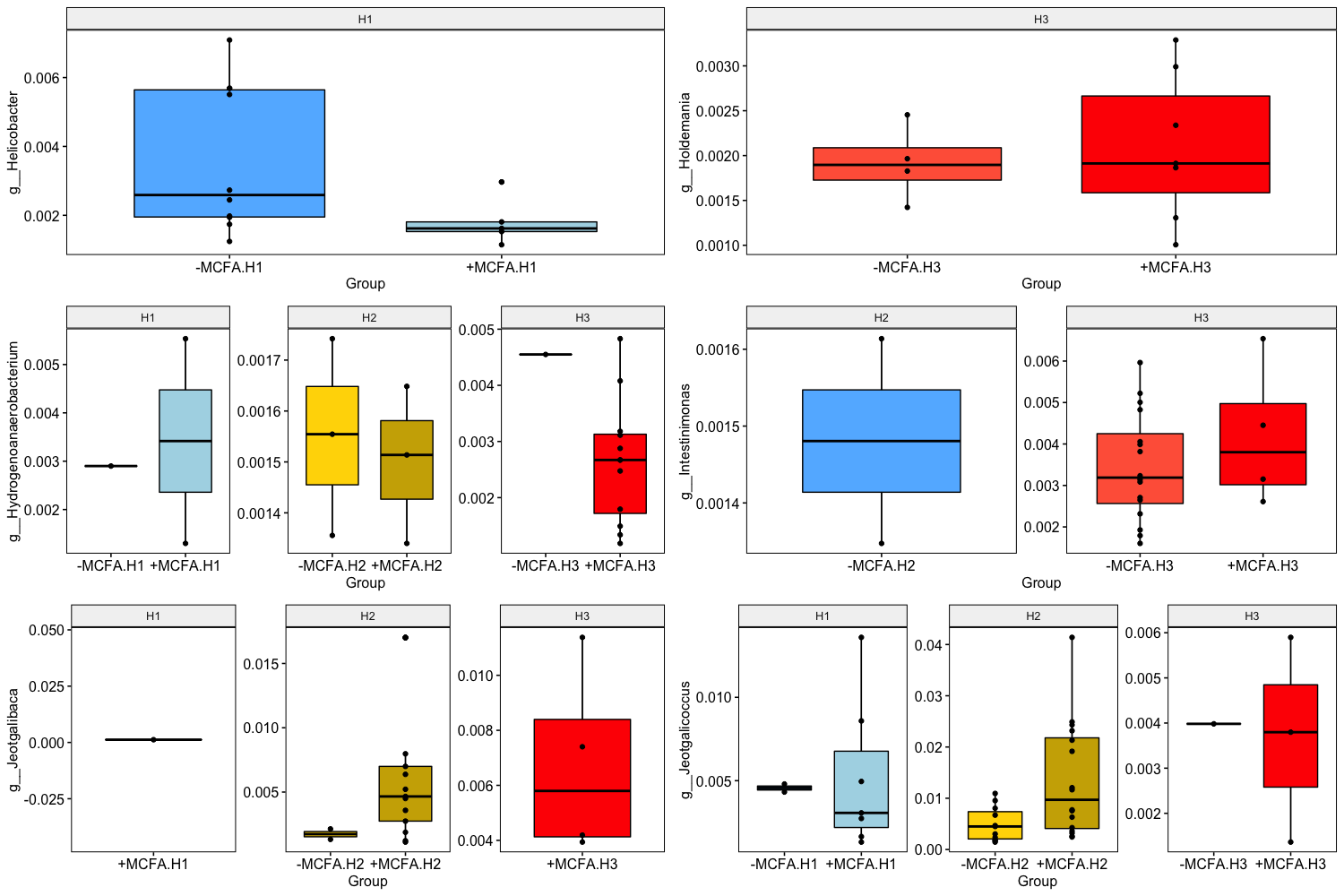
**

**
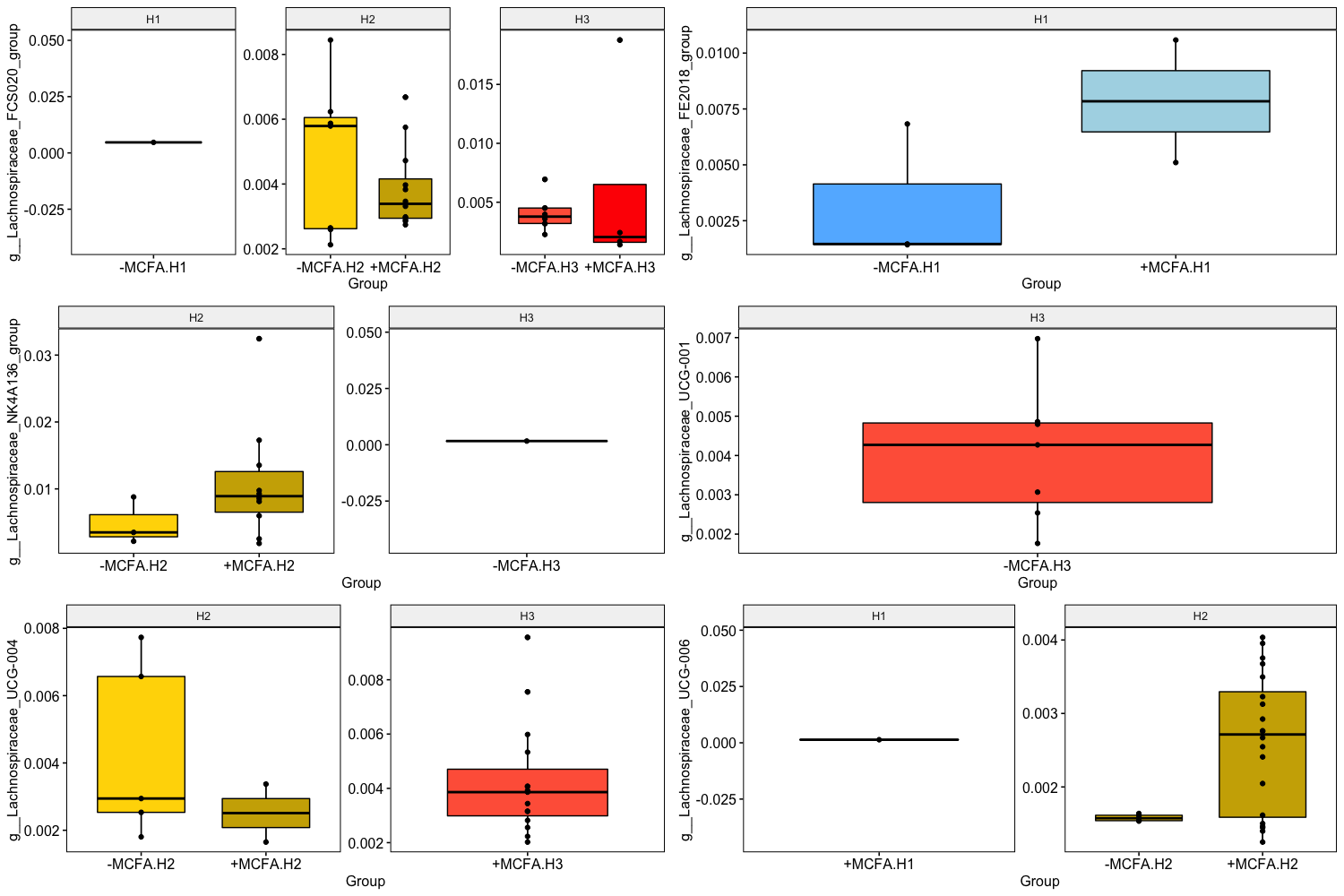

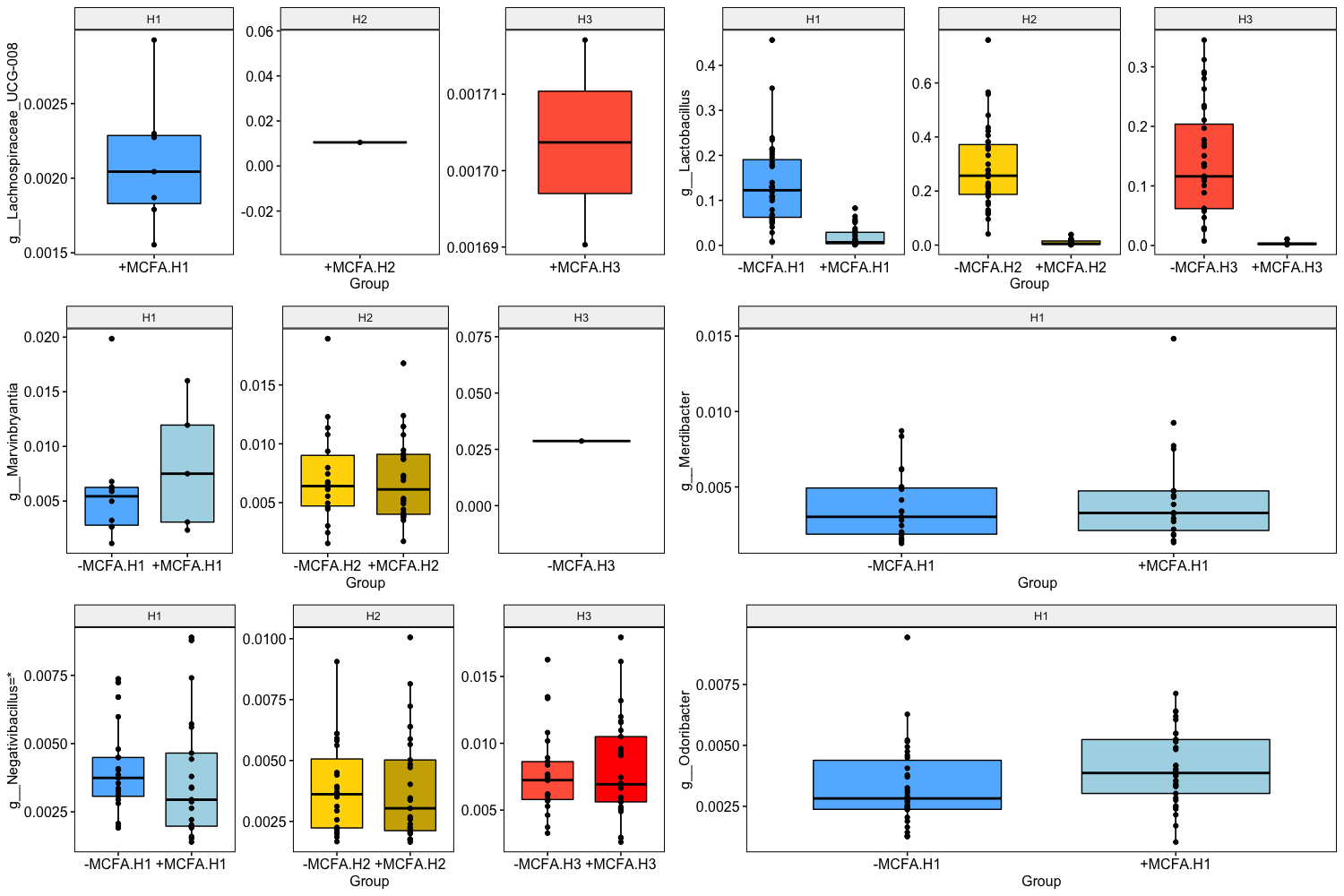
**

**
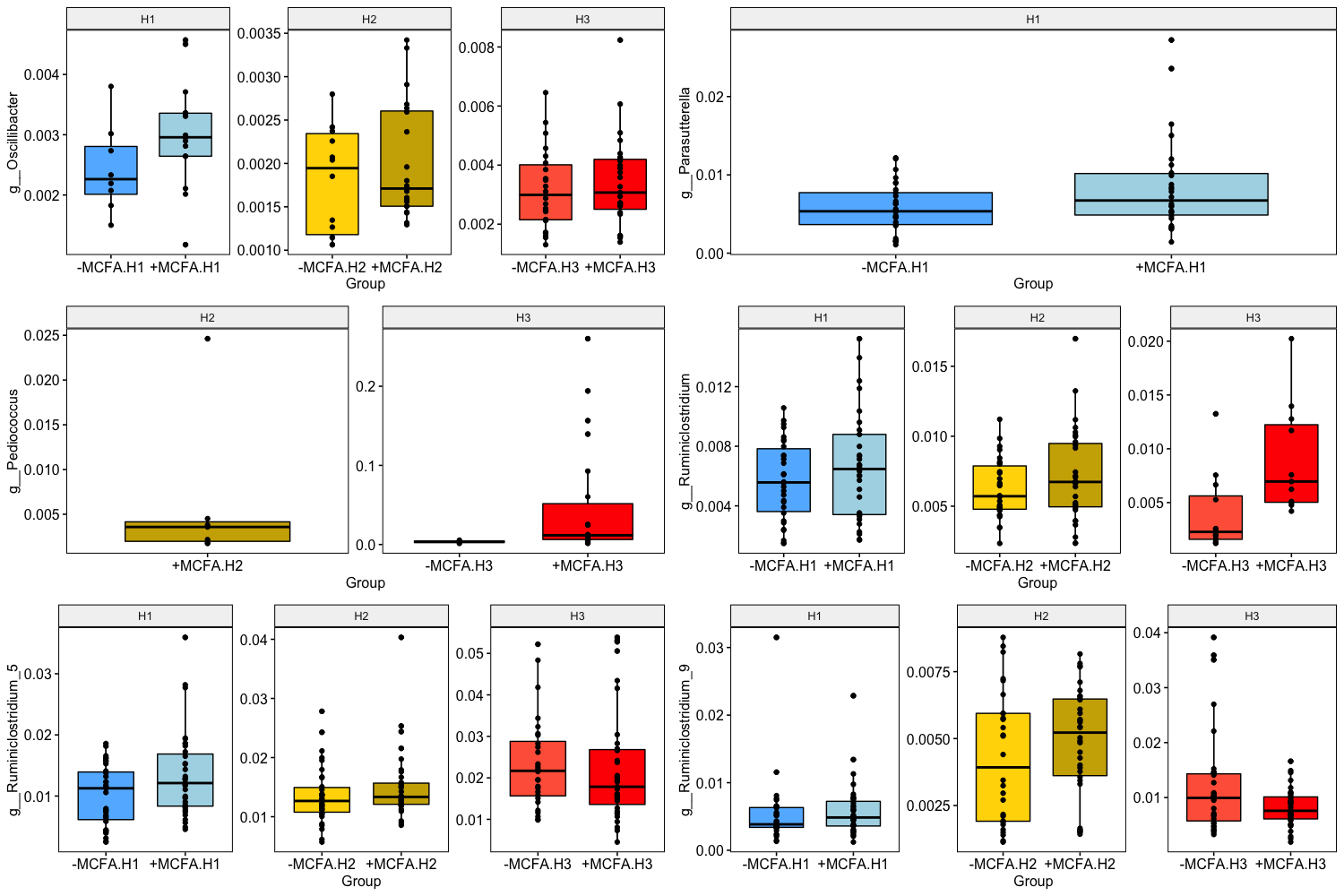

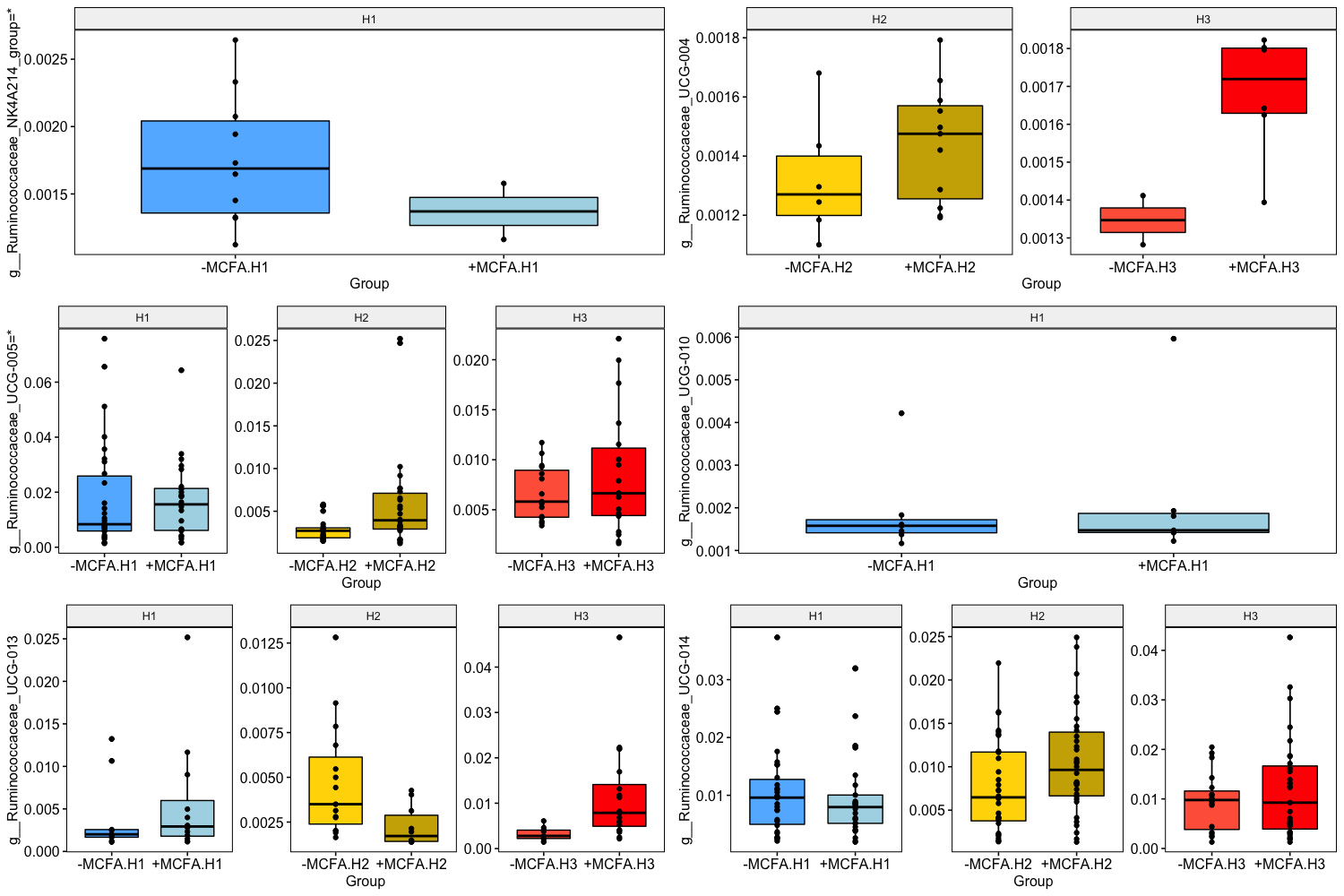
**

**
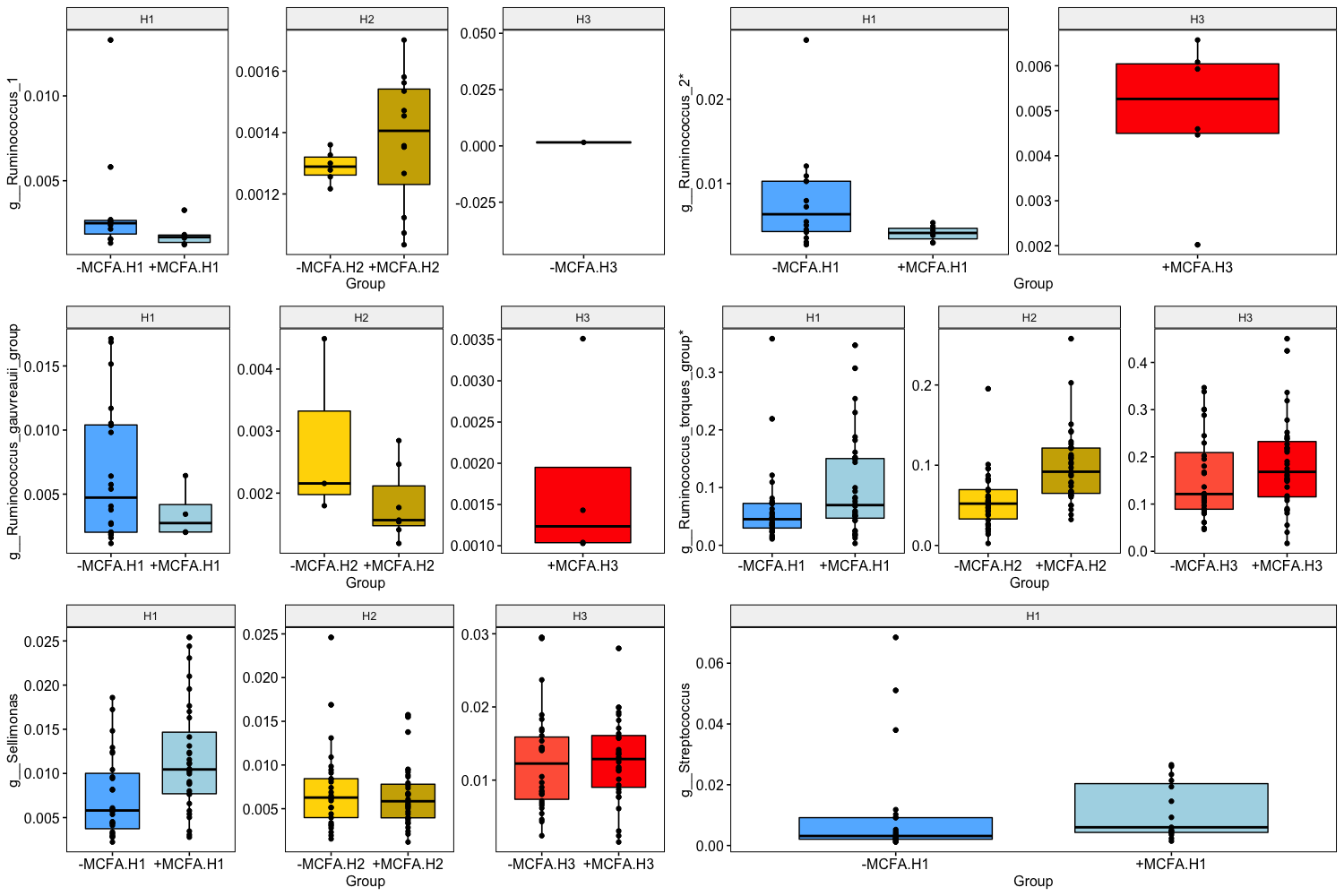

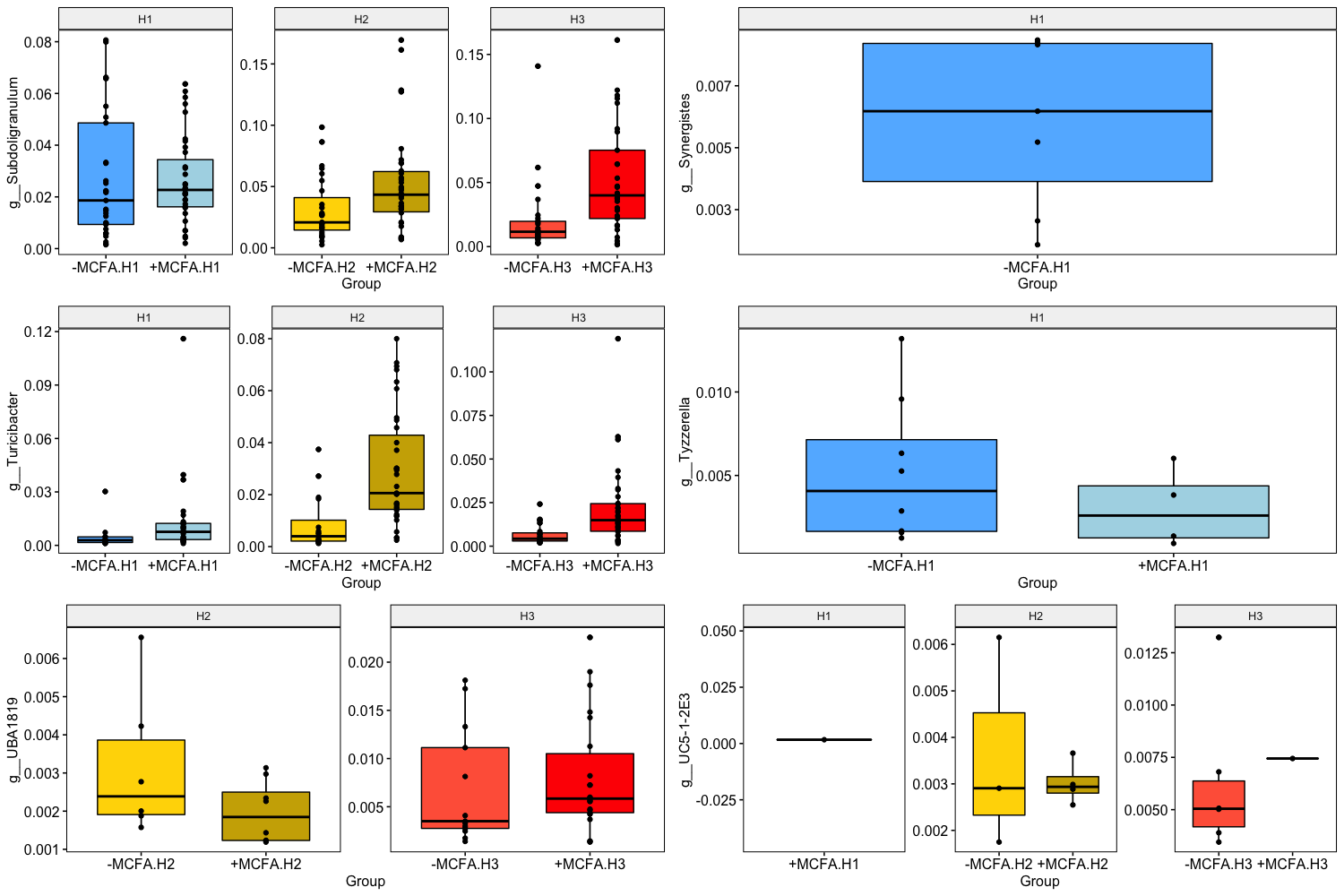
**


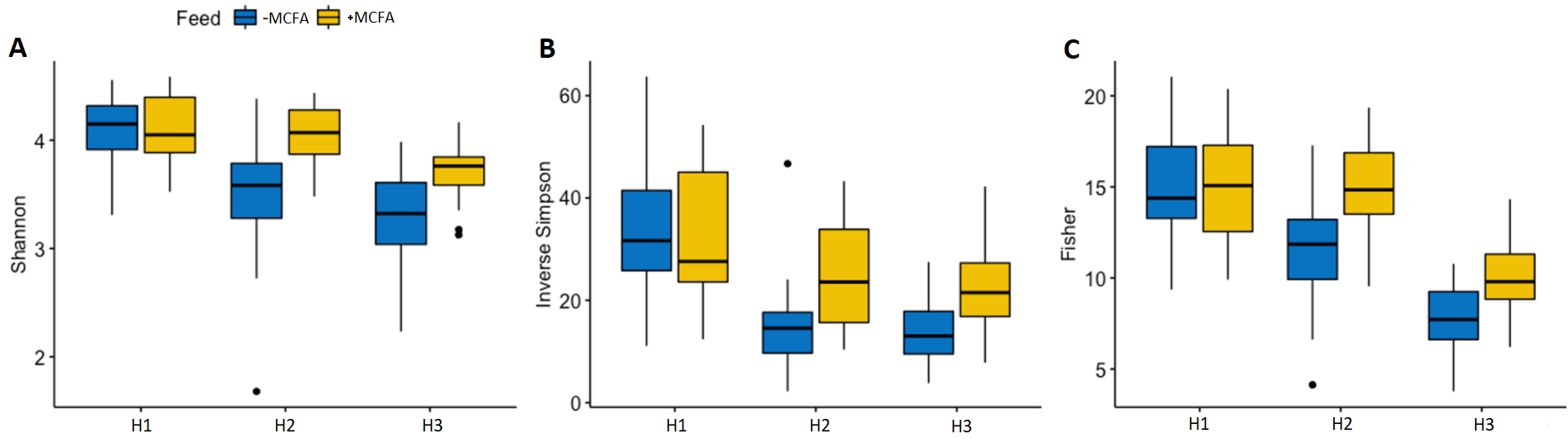
**Figure S3.** **Alpha diversity based on Shannon, Inverse Simpson and Fisher showed the same trend as the phylogenetic diversity.**

Shannon, Inverse Simpson and Fisher showed no difference between –MCFA and +MCFA fed broilers in H1 (A. *p* = 0.735, B. *p* = 0.651, C. *p* = 1.000), but in H2 and H3 +MCFA fed broilers have an increased alpha diversity (*p* > 0.001).

**Figure S4.** **Principle Coordinates Analysis based on Bray-Curtis, Jaccard, Unweighted UniFrac and Weighted UniFrac distance matrices.**

**a** Based on Bray-curtis distance the factor feed explained around 6% of variation in microbiota composition across all housing conditions. **b** Based on Jaccard distance the factor feed explained around 4% across all housing units. **c** Based on unweighted UniFrac distance the factor feed explained also 4% across all housing units, however, besides a location effect, also a dispersion effect was found (F=24. *P* =0.001). **d** Based on weighted UniFrac distance the factor feed explained 10% of the variation across all housing units. Table below shows effects per housing condition.


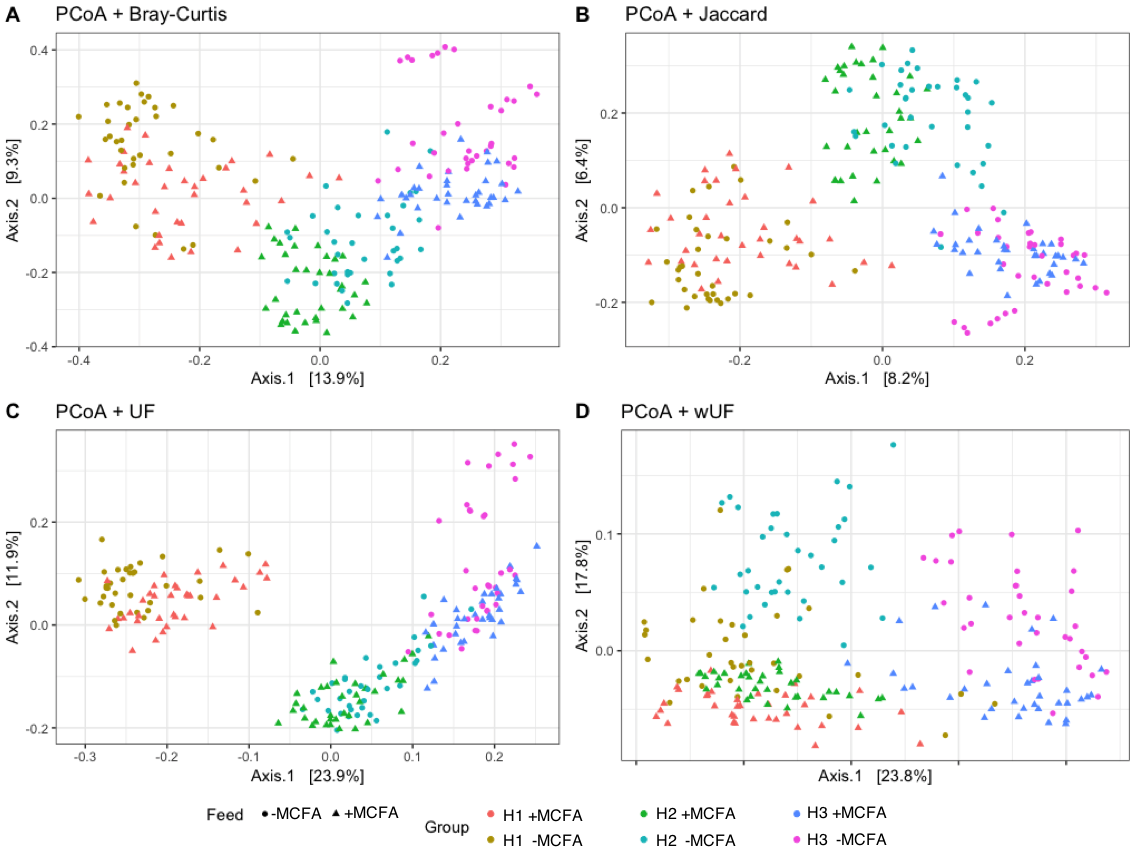


| Distance | H1 | H2 | H3 |
| --- | --- | --- | --- |
| Bray-curtis | Feed. R2=0.073. F=5.3. *P*=1e-04  Cage. R2=0.247. F=2.2. *P=*1e-04  Beta dis. *P*=0.227 / 0.233 | Feed. R2=0.154. F=12.4. *P*=1e-04  Cage. R2=0.433. F=5.1. *P=*1e-04  Beta dis. *P*=0.134 / 0.246 | Feed. R2=0.131. F=10.3. *P*=1e-04  Cage. R2=0.564. F=8.6. *P=*1e-04  Beta dis. *P*=0.171/ 0.119 |
| Jaccard | Feed. R2=0.048. F=3.4. *P*=1e-04  Cage. R2=0.204. F=1.7. *P=*1e-04  Beta dis. *P*=0.246 / 0.3 | Feed. R2=0.105. F=8. *P*=1e-04  Cage. R2=0.342. F=3.5. *P=*1e-04  Beta dis. *P*=0.125 / 0.358 | Feed. R2=0.088. F=6.6. *P*=1e-04  Cage. R2=0.441. F=5.2. *P=*1e-04  Beta dis. *P*=0.284 / 0.180 |
| Unweighted UniFrac | Feed. R2=0.087. F=6.5. *P*=1e-04  Cage. R2=0.228. F=1.9. *P=*1e-04  Beta dis. *P*=0.411 / 0.486 | Feed. R2=0.10. F=7.3. *P*=1e-04  Cage. R2=0.317. F=2.2. *P=*1e-04  Beta dis. *P*=0.014* / 0.568 | Feed. R2=0.130. F=10.2. *P*=1e-04  Cage. R2=0.643. F=12. *P=*1e-04  Beta dis. *P*=0.001*** / 0.573 |
| Weighted UniFrac | Feed. R2=0.113. F=8.6. *P*=1e-04  Cage. R2=0.257. F=2.3. *P=*1e-04  Beta dis. *P*=0.939 / 0.218 | Feed. R2=0.278. F=26.1. *P*=1e-04  Cage. R2=0.536. F=7.7. *P=*1e-04  Beta dis. *P*=0.534 / 0.094 | Feed. R2=0.127. F=9.9. *P*=1e-04  Cage. R2=0.552. F=8.2. *P=*1e-04  Beta dis. *P*=0.006*** / 0.424 |

**Figure S5. Percentage of microbial taxa shared between pens within a housing condition.**

**a-c** Percentage of OTUs shared between pens, no correlation between distance and the shared percentage of OTUs (Pearson’s correlation) **d-f** Percentage of genera shared between pens, no correlation between distance and the shared percentage of genera (Pearson’s correlation).

Figure S6 **Housing conditions (H1-H3).**

**a.** H1 is a research facility at Cargill Animal Nutrition Innovation Center, consisting of standard grow-out pens used for broiler feed experiments. Between the pens steel mesh panels were used as dividers, and the raised metal floor was covered with paper and a 2cm layer of wood shavings. **b** H2 are floor pens at the Faculty of Veterinary Medicine of Utrecht University. Adjacent pens were separated by solid wooden panels with only a mesh panel at the front of the pen. A single pen was present in four corners, and in the middle six pens were connected with each other. The floor of each pen was covered with a 2cm layer of wood shavings. **c** H3 are negative pressure HEPA filtered isolators. The floor consisted of a box filled with wood shavings to the same amount as in H1 and H2. The other 0.58m^2^ consisted of a plastic mesh floor.


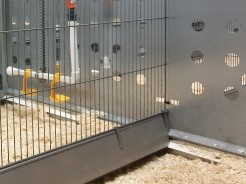

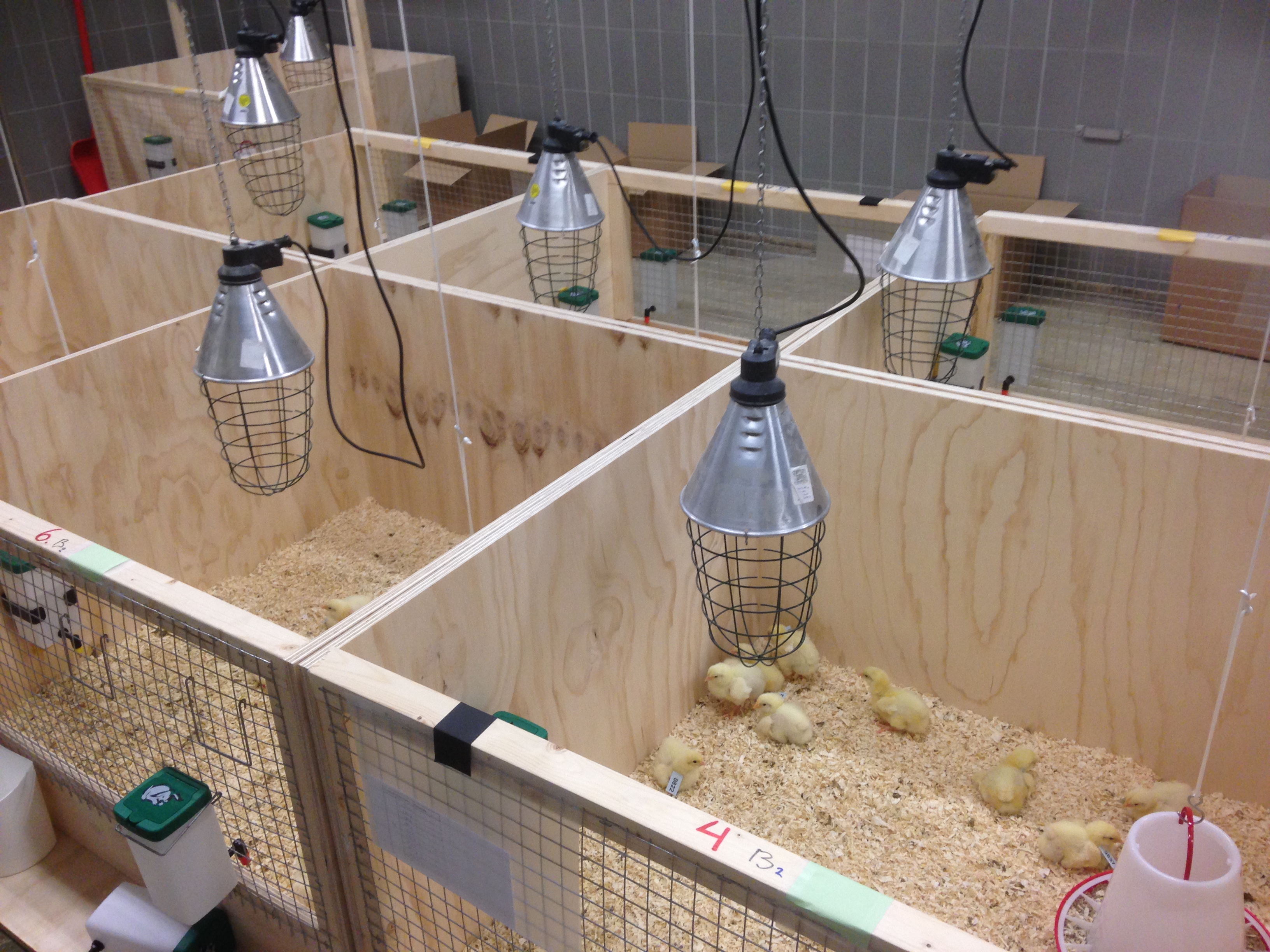

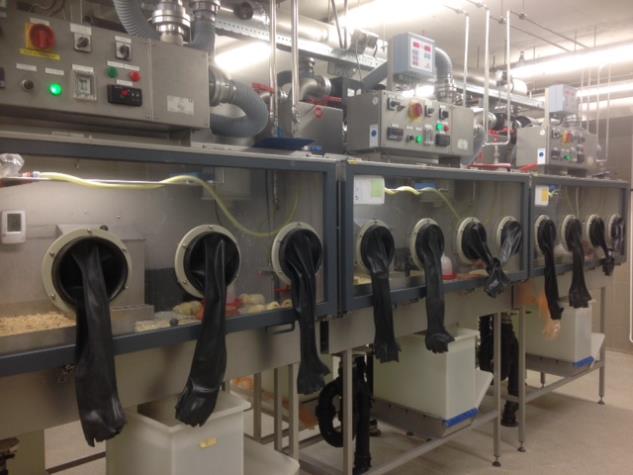


a. Housing condition 1 (H1) b. Housing condition 2 (H2) c. Housing condition 3 (H3)

**Table S1** Cecal microbiota composition at family level per housing condition and nutritional **intervention.** The relative abundance of all families, per housing condition and nutritional intervention (n= 35 broilers per group. - = not detected, and 0.00 = relative abundance below 0.004, numbers are in proportion (0-1)).

|  | H1 +MCFA | H1 -MCFA | H2 +MCFA | H2 -MCFA | H3 +MCFA | H3 -MCFA |
| --- | --- | --- | --- | --- | --- | --- |
| f__Acidaminococcaceae | - | 0.00 | - | - | - | - |
| f__Actinomycetales_bacterium_JB111 | - | - | 0.00 | - | - | - |
| f__Aerococcaceae | 0.00 | 0.00 | 0.00 | 0.00 | 0.00 | - |
| f__Akkermansiaceae | 0.01 | 0.00 | - | - | - | - |
| f__Anaeroplasmataceae | 0.00 | - | 0.00 | 0.00 | - | - |
| f__Bacillaceae | 0.00 | 0.00 | 0.01 | 0.00 | 0.02 | 0.01 |
| f__Bacteroidaceae | 0.03 | 0.05 | - | - | - | 0.00 |
| f__Barnesiellaceae | 0.01 | 0.01 | - | - | - | - |
| f__Bifidobacteriaceae | - | - | 0.00 | - | - | 0.00 |
| f__Brevibacteriaceae | 0.00 | 0.00 | 0.00 | 0.00 | - | 0.00 |
| f__Burkholderiaceae | 0.01 | 0.00 | 0.00 | 0.00 | - | - |
| f__Carnobacteriaceae | 0.00 | - | 0.00 | 0.00 | 0.00 | - |
| f__Christensenellaceae | 0.00 | 0.01 | 0.00 | 0.00 | 0.00 | - |
| f__Clostridiaceae_1 | 0.00 | 0.00 | - | 0.00 | - | 0.00 |
| f__Clostridiales_vadinBB60_group | 0.03 | 0.01 | 0.02 | 0.02 | 0.02 | 0.03 |
| f__Corynebacteriaceae | 0.00 | 0.00 | 0.02 | 0.01 | 0.00 | 0.00 |
| f__Defluviitaleaceae | 0.00 | 0.00 | 0.00 | 0.00 | 0.00 | 0.00 |
| f__Dermabacteraceae | 0.00 | 0.00 | 0.03 | 0.01 | 0.00 | 0.00 |
| f__Desulfovibrionaceae | 0.00 | 0.00 | - | - | - | - |
| f__Dietziaceae | - | - | 0.00 | 0.00 | - | - |
| f__Dysgonomonadaceae | - | - | - | - | - | 0.00 |
| f__Eggerthellaceae | 0.00 | 0.00 | 0.00 | 0.00 | 0.00 | 0.00 |
| f__Elusimicrobiaceae | - | 0.00 | - | - | - | - |
| f__Enterobacteriaceae | 0.02 | 0.00 | 0.02 | 0.00 | 0.02 | 0.01 |
| f__Enterococcaceae | 0.00 | 0.00 | 0.00 | 0.00 | 0.00 | 0.00 |
| f__Erysipelotrichaceae | 0.04 | 0.03 | 0.06 | 0.02 | 0.07 | 0.05 |
| f__Eubacteriaceae | 0.00 | 0.00 | - | - | - | - |
| f__Family_XIII | 0.00 | 0.00 | 0.00 | 0.00 | 0.00 | 0.00 |
| f__Healicobacteraceae | 0.00 | 0.00 | - | - | - | - |
| f__Lachnospiraceae | 0.38 | 0.35 | 0.40 | 0.32 | 0.58 | 0.59 |
| f__Lactobacillaceae | 0.02 | 0.14 | 0.01 | 0.32 | 0.04 | 0.14 |
| f__Marinifilaceae | 0.00 | 0.01 | - | - | - | - |
| f__Microbacteriaceae | - | - | 0.00 | - | - | - |
| f__Micrococcaceae | 0.00 | 0.00 | 0.00 | 0.00 | - | 0.00 |
| f__Peptococcaceae | 0.00 | 0.00 | 0.00 | 0.00 | - | - |
| f__Peptostreptococcaceae | 0.05 | 0.02 | 0.03 | 0.05 | 0.05 | 0.05 |
| f__Puniceicoccaceae | - | 0.00 | - | - | - | - |
| f__Rikenellaceae | 0.02 | 0.02 | - | - | - | 0.00 |
| f__Ruminococcaceae | 0.34 | 0.29 | 0.37 | 0.24 | 0.18 | 0.11 |
| f__Saccharimonadaceae | - | 0.00 | - | - | - | - |
| f__Staphylococcaceae | 0.00 | 0.00 | 0.01 | 0.00 | 0.00 | 0.00 |
| f__Streptococcaceae | 0.01 | 0.01 | - | - | - | - |
| f__Synergistaceae | - | 0.00 | - | - | - | - |
| f__uncultured_Firmicutes_bacterium | 0.00 | - | - | - | - | - |
| f__unknown | 0.03 | 0.02 | 0.01 | 0.01 | 0.01 | 0.00 |
| f__Veillonellaceae | - | - | - | - | - | 0.00 |

**Table S2:** **Abundance testing for genera that were significantly different between the chicks on +MCFA or -MCFA feed and between the housing conditions.**

The results are based on differences of relative abundance tested with Wilcoxon rank-sum test. Adjusted P-values (0.05) are corrected for multiple testing with BH. The relative abundance and variation between groups of those genera are presented in Figure S3.

| **Housing condition 1** | Adjusted P <0.05 | Change in relative abundance (percent points) |
| --- | --- | --- |
| f__Lactobacillaceae;g__Lactobacillus | 1.02e-08 | 11.94% |
| f__Peptostreptococcaceae;g__ | 1.15e-04 | -2.69% |
| f__Bacteroidaceae;g__Bacteroides | 3.03e-02 | 2.24% |
| f__Enterobacteriaceae;g__Escherichia-Shigella | 1.18e-07 | -1.92% |
| f__Erysipelotrichaceae;g__Turicibacter | 1.15e-04 | -0.91% |
| f__Clostridiales_vadinBB60_group;g__uncultured_bacterium | 3.96e-02 | -0.87% |
| f__Lachnospiraceae;g__[Eubacterium]_hallii_group | 7.43e-03 | 0.74% |
| f__Lachnospiraceae;g__Sellimonas | 2.60e-02 | -0.49% |
| f__Marinifilaceae;g__Butyricimonas | 1.18e-07 | 0.43% |
| f__Lachnospiraceae;g__[Ruminococcus]_gauvreauii_group | 1.29e-03 | 0.34% |
| f__Bacillaceae;g__Bacillus | 4.29e-02 | -0.22% |
| f__Lachnospiraceae;g__Anaerostipes | 4.89e-02 | -0.19% |
| f__Rikenellaceae;g__ | 4.89e-02 | 0.14% |
| f__Synergistaceae;g__Synergistes | 4.89e-02 | 0.12% |
| f__Lachnospiraceae;g__Lachnospiraceae_UCG-008 | 4.89e-02 | -0.04% |
| **Housing condition 2** |  |  |
| f__Lactobacillaceae;g__Lactobacillus | 6.19e-11 | 27.93% |
| f__Ruminococcaceae;g__Faecalibacterium | 4.98e-02 | -6.26% |
| f__Lachnospiraceae;g__[Ruminococcus]_torques_group | 7.67e-05 | -4.35% |
| f__Ruminococcaceae;g__Subdoligranulum | 8.06e-03 | -2.63% |
| f__Erysipelotrichaceae;g__Turicibacter | 1.76e-08 | -2.56% |
| f__Lachnospiraceae;g__Fusicatenibacter | 2.32e-04 | -1.76% |
| f__Enterobacteriaceae;g__Escherichia-Shigella | 1.83e-09 | -1.55% |
| f__Erysipelotrichaceae;g__Erysipelatoclostridium | 2.27e-03 | -1.03% |
| f__Ruminococcaceae;g__uncultured | 9.62e-03 | -0.54% |
| f__Lachnospiraceae;g__Anaerostipes | 1.26e-03 | 0.38% |
| f__Lachnospiraceae;g__[Eubacterium]_hallii_group | 2.29e-02 | 0.37% |
| f__Defluviitaleaceae;g__Defluviitaleaceae_UCG-011 | 3.48e-03 | -0.23% |
| f__Carnobacteriaceae;g__Jeotgalibaca | 8.59e-03 | -0.22% |
| f__Ruminococcaceae;g__[Eubacterium]_coprostanoligenes_group | 4.98e-02 | -0.16% |
| f__Lachnospiraceae;g__Lachnospiraceae_UCG-006 | 5.55e-04 | -0.13% |
| f__Aerococcaceae;g__Aerosphaera | 7.34e-03 | -0.13% |
| f__Lactobacillaceae;g__Pediococcus | 3.37e-02 | -0.12% |
| f__Lactobacillaceae;g__ | 3.37e-02 | 0.09% |
| f__Micrococcaceae;g__Glutamicibacter | 2.78e-02 | -0.06% |
| **Housing condition 3** |  |  |
| f__Lactobacillaceae;g__Lactobacillus | 7.69e-12 | 14.09% |
| f__Lachnospiraceae;g__Blautia | 2.60e-05 | 9.75% |
| f__Lachnospiraceae;g__uncultured | 5.35e-03 | -4.17% |
| f__Lactobacillaceae;g__Pediococcus | 1.71e-05 | -3.00% |
| f__Ruminococcaceae;g__Subdoligranulum | 1.81e-02 | -2.84% |
| f__Erysipelotrichaceae;g__Erysipelatoclostridium | 1.98e-04 | -2.15% |
| f__Erysipelotrichaceae;g__[Clostridium]_innocuum_group | 3.65e-02 | 2.15% |
| f__Erysipelotrichaceae;g__Turicibacter | 6.80e-05 | -1.64% |
| f__Enterobacteriaceae;g__Escherichia-Shigella | 2.51e-02 | -0.98% |
| f__Lachnospiraceae;g__GCA-900066575 | 7.44e-03 | -0.92% |
| f__Lachnospiraceae;g__Eisenbergiella | 1.81e-02 | -0.78% |
| f__Ruminococcaceae;g__Ruminococcaceae_UCG-013 | 7.79e-03 | -0.57% |
| f__Ruminococcaceae;g__Ruminococcaceae_UCG-014 | 2.60e-02 | -0.54% |
| o__Clostridiales;f__ | 7.79e-03 | -0.38% |
| o__Mollicutes_RF39;f__uncultured_bacterium | 5.35e-03 | -0.24% |
| f__Bacteroidaceae;g__Bacteroides | 2.60e-02 | 0.23% |
| f__Lachnospiraceae;g__Lachnospiraceae_UCG-004 | 3.64e-04 | -0.18% |
| f__Enterococcaceae;g__Enterococcus | 2.22e-02 | 0.17% |
| f__Corynebacteriaceae;g__Corynebacterium_1 | 2.60e-02 | -0.17% |
| f__Lactobacillaceae;g__ | 1.28e-02 | 0.15% |
| f__Ruminococcaceae;g__Intestinimonas | 1.92e-02 | 0.11% |
| f__Aerococcaceae;g__Aerococcus | 2.60e-02 | -0.09% |
| f__Ruminococcaceae;g__Ruminococcus_2 | 4.63e-02 | -0.08% |
| f__Lachnospiraceae;g__Lachnospiraceae_UCG-001 | 2.60e-02 | 0.08% |
| f__Ruminococcaceae;g__Hydrogenoanaerobacterium | 1.10e-02 | -0.08% |
| **Housing condition 1 vs Housing condition 2** |  |  |
| f__Bacteroidaceae;g__Bacteroides | 2.76e-25 | 4.20% |
| f__Lachnospiraceae;g__uncultured | 5.84e-08 | -3.35% |
| f__Lachnospiraceae;g__Blautia | 7.01e-05 | 2.85% |
| f__Dermabacteraceae;g__Brachybacterium | 2.49e-09 | -2.25% |
| f__Lachnospiraceae;g__CHKCI001 | 3.74e-11 | 2.17% |
| f__Rikenellaceae;g__Alistipes | 2.76e-25 | 1.90% |
| f__Lachnospiraceae;g__Fusicatenibacter | 0.013 | 1.79% |
| f__Lachnospiraceae;g__Eisenbergiella | 3.95e-07 | -1.37% |
| f__Corynebacteriaceae;g__Corynebacterium_1 | 6.69e-05 | -1.15% |
| f__Lachnospiraceae;g__GCA-900066575 | 4.90e-13 | -1.05% |
| f__Erysipelotrichaceae;g__Turicibacter | 0.002 | -1.05% |
| f__Ruminococcaceae;g__Ruminococcaceae_UCG-005 | 6.69e-05 | 1.01% |
| f__Ruminococcaceae;g__Butyricicoccus | 1.16e-11 | 0.87% |
| f__Akkermansiaceae;g__Akkermansia | 1.18e-06 | 0.82% |
| f__Ruminococcaceae;g__uncultured | 0.001 | 0.77% |
| f__Barnesiellaceae;g__Barnesiella | 1.10e-19 | 0.71% |
| o__Clostridiales;f__ | 2.18e-05 | 0.69% |
| f__Streptococcaceae;g__Streptococcus | 7.51e-12 | 0.59% |
| f__Burkholderiaceae;g__Parasutterella | 2.54e-18 | 0.56% |
| o__Mollicutes_RF39;f__uncultured_bacterium | 0.001 | 0.55% |
| f__Christensenellaceae;g__Christensenellaceae_R-7_group | 5.21e-05 | 0.48% |
| f__Clostridiales_vadinBB60_group;g__ | 1.51e-08 | 0.47% |
| f__Ruminococcaceae;g__Fournierella | 1.64e-08 | 0.38% |
| f__Ruminococcaceae;g__[Eubacterium]_coprostanoligenes_group | 9.83e-12 | -0.35% |
| f__Marinifilaceae;g__Odoribacter | 3.91e-22 | 0.33% |
| f__Staphylococcaceae;g__Jeotgalicoccus | 0.001 | -0.32% |
| f__Lachnospiraceae;g__Marvinbryantia | 3.41e-06 | -0.30% |
| f__Clostridiales_vadinBB60_group;g__uncultured_bacterium | 0.046 | -0.30% |
| f__Anaeroplasmataceae;g__Anaeroplasma | 0.002 | -0.28% |
| f__Marinifilaceae;g__Butyricimonas | 1.64e-10 | 0.26% |
| f__Lachnospiraceae;g__Coprococcus_3 | 1.48e-07 | 0.26% |
| f__Eggerthellaceae;g__CHKCI002 | 1.58e-17 | 0.25% |
| f__Peptococcaceae;g__ | 2.15e-15 | 0.24% |
| f__Defluviitaleaceae;g__Defluviitaleaceae_UCG-011 | 9.97e-12 | -0.24% |
| f__Ruminococcaceae;g__Ruminiclostridium_5 | 0.014 | -0.24% |
| f__Erysipelotrichaceae;g__Merdibacter | 1.37e-12 | 0.23% |
| f__Lachnospiraceae;g__Anaerostipes | 4.10e-4 | -0.22% |
| f__Ruminococcaceae;g__Ruminococcus_2 | 3.89e-06 | 0.20% |
| f__Desulfovibrionaceae;g__Bilophila | 1.58e-17 | 0.19% |
| f__Eggerthellaceae;g__Gordonibacter | 5.64e-16 | -0.19% |
| f__uncultured_bacterium;g | 3.89e-06 | 0.19% |
| f__Bacillaceae;g__Bacillus | 0.001 | -0.18% |
| o__Mollicutes_RF39;f__uncultured_bacterium_ | 1.06e-09 | 0.18% |
| f__Lachnospiraceae;g__[Ruminococcus]_gauvreauii_group | 0.004 | 0.18% |
| f__Lachnospiraceae;g__Lachnospiraceae_NK4A136_group | 0.001 | -0.18% |
| o__Mollicutes_RF39;f__ | 1.42e-06 | 0.13% |
| f__Carnobacteriaceae;g__Jeotgalibaca | 0.001 | -0.12% |
| f__Lachnospiraceae;g__Lachnospiraceae_FCS020_group | 6.69e-05 | -0.11% |
| f__Barnesiellaceae;g__ | 0.026 | 0.09% |
| o__Gastranaerophilales;f__uncultured_bacterium | 7.53e-06 | 0.09% |
| f__Rikenellaceae;g__ | 1.64e-4 | 0.09% |
| f__Dietziaceae;g__Dietzia | 1.64e-4 | -0.09% |
| f__Lachnospiraceae;g__Lachnospiraceae_UCG-006 | 1.92e-06 | -0.08% |
| f__Barnesiellaceae;g__Coprobacter | 3.89e-06 | 0.08% |
| f__Lachnospiraceae;g__Shuttleworthia | 0.001 | -0.08% |
| f__Lachnospiraceae;g__Tyzzerella | 0.001 | 0.08% |
| f__Helicobacteraceae;g__Helicobacter | 1.61e-4 | 0.06% |
| f__Aerococcaceae;g__Aerosphaera | 0.003 | -0.06% |
| f__Aerococcaceae;g__Aerococcus | 0.033 | -0.06% |
| f__Lactobacillaceae;g__Pediococcus | 0.015 | -0.06% |
| f__Synergistaceae;g__Synergistes | 0.015 | 0.06% |
| f__Ruminococcaceae;g__UBA1819 | 2.93e-4 | -0.05% |
| f__Ruminococcaceae;g__Candidatus_Soleaferrea | 0.003 | 0.05% |
| f__Ruminococcaceae;g__DTU089 | 0.023 | -0.04% |
| f__Ruminococcaceae;g__Ruminococcaceae_UCG-010 | 2.91e-4 | 0.04% |
| f__Lachnospiraceae;g__Lachnospiraceae_UCG-004 | 0.015 | -0.04% |
| o__Gastranaerophilales;f__ | 0.001 | 0.04% |
| f__Micrococcaceae;g__Glutamicibacter | 0.002 | -0.04% |
| f__Lachnospiraceae;g__Lachnospiraceae_FE2018_group | 0.045 | 0.04% |
| f__Ruminococcaceae;g__Flavonifractor | 0.033 | -0.03% |
| f__Ruminococcaceae;g__Ruminococcaceae_UCG-004 | 5.21e-05 | -0.03% |
| f__Ruminococcaceae;g__Ruminococcaceae_NK4A214_group | 0.001 | 0.03% |
| f__Peptococcaceae;g__uncultured | 0.024 | -0.03% |
| f__Eggerthellaceae;g__ | 0.002 | 0.02% |
| f__Lachnospiraceae;g__Tyzzerella_3 | 0.045 | 0.02% |
| f__Lachnospiraceae;g__Roseburia | 0.026 | 0.02% |
| o__Izimaplasmatales;f__ | 0.045 | 0.01% |
| **Housing condition 1 vs Housing condition 3** |  |  |
| f__Ruminococcaceae;g__Faecalibacterium | 5.63e-22 | 17.09% |
| f__Lachnospiraceae;g__Blautia | 8.95e-09 | -9.78% |
| f__Lachnospiraceae;g__[Ruminococcus]_torques_group | 4.35e-06 | -7.55% |
| f__Lachnospiraceae;g__Eisenbergiella | 4.59e-05 | -4.40% |
| f__Bacteroidaceae;g__Bacteroides | 5.58e-23 | 4.08% |
| f__Lachnospiraceae;g__uncultured | 0.001 | -3.78% |
| f__Lachnospiraceae;g__Fusicatenibacter | 1.74e-10 | 2.98% |
| f__Peptostreptococcaceae;g__ | 0.035 | -1.91% |
| f__Rikenellaceae;g__Alistipes | 8.31e-25 | 1.90% |
| f__Ruminococcaceae;g__uncultured | 1.09e-13 | 1.54% |
| f__Lactobacillaceae;g__Pediococcus | 4.59e-07 | -1.52% |
| f__Erysipelotrichaceae;g__Erysipelatoclostridium | 1.08e-06 | -1.50% |
| f__Lachnospiraceae;g__CHKCI001 | 2.90e-07 | 1.44% |
| f__Clostridiales_vadinBB60_group;g__uncultured_bacterium | 0.004 | -1.40% |
| f__Erysipelotrichaceae;g__[Clostridium]_innocuum_group | 1.36e-10 | -1.35% |
| f__Ruminococcaceae;g__Ruminococcaceae_UCG-005 | 3.71e-05 | 0.96% |
| o__Mollicutes_RF39;f__uncultured_bacterium | 1.52e-15 | 0.95% |
| f__Ruminococcaceae;g__Ruminiclostridium_5 | 1.10e-05 | -0.88% |
| f__Akkermansiaceae;g__Akkermansia | 8.85e-07 | 0.82% |
| f__Christensenellaceae;g__Christensenellaceae_R-7_group | 7.46e-20 | 0.77% |
| f__Barnesiellaceae;g__Barnesiella | 7.46e-20 | 0.71% |
| f__Erysipelotrichaceae;g__Turicibacter | 3.01e-4 | -0.64% |
| f__Clostridiales_vadinBB60_group;g__ | 9.42e-18 | 0.61% |
| f__Streptococcaceae;g__Streptococcus | 5.41e-12 | 0.59% |
| f__Burkholderiaceae;g__Parasutterella | 1.62e-18 | 0.56% |
| f__Ruminococcaceae;g__Butyricicoccus | 1.91e-4 | 0.50% |
| f__Ruminococcaceae;g__Flavonifractor | 1.15e-14 | -0.48% |
| f__Ruminococcaceae;g__Ruminiclostridium_9 | 1.68e-08 | -0.47% |
| f__Ruminococcaceae;g__Fournierella | 1.30e-08 | 0.38% |
| f__Ruminococcaceae;g__UBA1819 | 1.36e-10 | -0.38% |
| f__Ruminococcaceae;g__Negativibacillus | 6.01e-07 | -0.38% |
| f__Ruminococcaceae;g__Ruminiclostridium | 1.38e-09 | 0.36% |
| f__Marinifilaceae;g__Odoribacter | 3.99e-22 | 0.33% |
| f__Eggerthellaceae;g__Gordonibacter | 3.55e-19 | -0.33% |
| f__Ruminococcaceae;g__[Eubacterium]_coprostanoligenes_group | 1.56e-08 | -0.32% |
| f__Lachnospiraceae;g__Sellimonas | 0.025 | -0.28% |
| f__Erysipelotrichaceae;g__uncultured_bacterium | 6.86e-09 | 0.27% |
| f__Lachnospiraceae;g__Coprococcus_3 | 1.36e-10 | 0.27% |
| f__Ruminococcaceae;g__ | 0.009 | 0.27% |
| f__Marinifilaceae;g__Butyricimonas | 1.36e-10 | 0.26% |
| f__Eggerthellaceae;g__CHKCI002 | 1.03e-17 | 0.25% |
| f__Peptococcaceae;g__ | 1.52e-15 | 0.24% |
| f__Erysipelotrichaceae;g__Merdibacter | 9.61e-13 | 0.23% |
| f__Ruminococcaceae;g__DTU089 | 2.98e-06 | -0.23% |
| f__Erysipelotrichaceae;g__ | 3.03e-05 | 0.21% |
| f__Lachnospiraceae;g__[Ruminococcus]_gauvreauii_group | 3.71e-05 | 0.20% |
| f__Desulfovibrionaceae;g__Bilophila | 1.03e-17 | 0.19% |
| f__uncultured_bacterium;g | 3.24e-06 | 0.19% |
| o__Mollicutes_RF39;f__uncultured_bacterium_ | 1.19e-09 | 0.18% |
| f__Ruminococcaceae;g__Ruminococcus_2 | 0.003 | 0.16% |
| f__Ruminococcaceae;g__Oscillibacter | 3.84e-06 | -0.15% |
| o__Mollicutes_RF39;f__ | 2.37e-07 | 0.13% |
| f__Ruminococcaceae;g__Intestinimonas | 5.85e-06 | -0.10% |
| f__Lachnospiraceae;g__Marvinbryantia | 0.001 | 0.10% |
| f__Lachnospiraceae;g__Anaerostipes | 0.010 | -0.10% |
| f__Barnesiellaceae;g__ | 0.025 | 0.09% |
| o__Gastranaerophilales;f__uncultured_bacterium | 5.85e-06 | 0.09% |
| f__Lachnospiraceae;g__Lachnospiraceae_UCG-004 | 1.38e-04 | -0.09% |
| f__Ruminococcaceae;g__Anaerotruncus | 0.004 | -0.09% |
| f__Rikenellaceae;g__ | 1.38e-04 | 0.09% |
| f__Barnesiellaceae;g__Coprobacter | 3.24e-06 | 0.08% |
| f__Lachnospiraceae;g__Tyzzerella | 0.001 | 0.08% |
| f__Lachnospiraceae;g__Lachnospiraceae_FCS020_group | 0.004 | -0.07% |
| f__Ruminococcaceae;g__Ruminococcus_1 | 1.54e-04 | 0.07% |
| f__Helicobacteraceae;g__Helicobacter | 1.38e-04 | 0.06% |
| f__Lachnospiraceae;g__UC5-1-2E3 | 0.049 | -0.06% |
| f__Lactobacillaceae;g__ | 0.049 | -0.06% |
| f__Synergistaceae;g__Synergistes | 0.014 | 0.06% |
| f__Brevibacteriaceae;g__Brevibacterium | 0.003 | 0.05% |
| f__Peptostreptococcaceae;g__Clostridioides | 0.043 | -0.04% |
| f__Ruminococcaceae;g__Ruminococcaceae_UCG-010 | 2.49e-04 | 0.04% |
| f__Lachnospiraceae;g__Lachnospiraceae_UCG-001 | 0.014 | -0.04% |
| f__Ruminococcaceae;g__Candidatus_Soleaferrea | 0.029 | 0.04% |
| f__Ruminococcaceae;g__Hydrogenoanaerobacterium | 0.018 | -0.04% |
| o__Gastranaerophilales;f__ | 0.001 | 0.04% |
| f__Lachnospiraceae;g__Lachnospiraceae_FE2018_group | 0.043 | 0.04% |
| f__Erysipelotrichaceae;g__Holdemania | 0.001 | -0.03% |
| f__Ruminococcaceae;g__Ruminococcaceae_NK4A214_group | 0.001 | 0.03% |
| f__Family_XIII;g__Family_XIII_UCG-001 | 0.001 | -0.03% |
| f__Anaeroplasmataceae;g__Anaeroplasma | 0.025 | 0.02% |
| f__Eggerthellaceae;g__ | 0.001 | 0.02% |
| f__Ruminococcaceae;g__Ruminococcaceae_UCG-004 | 0.008 | -0.02% |
| f__Peptococcaceae;g__uncultured | 0.008 | 0.02% |
| o__Izimaplasmatales;f__ | 0.043 | 0.01% |
| **Housing condition 2 vs Housing condition 3** |  |  |
| f__Ruminococcaceae;g__Faecalibacterium | 1.85e-22 | 19.65% |
| f__Lachnospiraceae;g__Blautia | 3.90e-16 | -12.63% |
| f__Lachnospiraceae;g__[Ruminococcus]_torques_group | 2.06e-06 | -7.93% |
| f__Lactobacillaceae;g__Lactobacillus | 0.002 | 7.58% |
| f__Dermabacteraceae;g__Brachybacterium | 1.19e-11 | 2.34% |
| f__Lactobacillaceae;g__Pediococcus | 0.001 | -1.46% |
| f__Erysipelotrichaceae;g__[Clostridium]_innocuum_group | 6.90e-10 | -1.34% |
| f__Lachnospiraceae;g__Fusicatenibacter | 3.23e-06 | 1.19% |
| f__Corynebacteriaceae;g__Corynebacterium_1 | 3.81e-3 | 1.16% |
| f__Erysipelotrichaceae;g__Erysipelatoclostridium | 0.002 | -1.10% |
| f__Enterobacteriaceae;g__Escherichia-Shigella | 0.044 | -0.77% |
| f__Ruminococcaceae;g__uncultured | 4.37e-08 | 0.77% |
| f__Ruminococcaceae;g__Ruminiclostridium_5 | 0.002 | -0.65% |
| f__Ruminococcaceae;g__Ruminiclostridium_9 | 5.03e-11 | -0.60% |
| o__Clostridiales;f__ | 6.04e-07 | -0.50% |
| f__Lachnospiraceae;g__Sellimonas | 4.56e-05 | -0.48% |
| f__Ruminococcaceae;g__Flavonifractor | 6.58e-11 | -0.45% |
| f__Lachnospiraceae;g__Marvinbryantia | 1.80e-12 | 0.41% |
| o__Mollicutes_RF39;f__uncultured_bacterium | 5.91e-10 | 0.40% |
| f__Lachnospiraceae;g__GCA-900066575 | 0.007 | 0.39% |
| f__Ruminococcaceae;g__Ruminiclostridium | 3.30e-09 | 0.38% |
| f__Ruminococcaceae;g__Butyricicoccus | 5.06e-07 | -0.37% |
| f__Staphylococcaceae;g__Jeotgalicoccus | 9.84e-06 | 0.36% |
| f__Ruminococcaceae;g__UBA1819 | 4.99e-05 | -0.33% |
| f__Ruminococcaceae;g__Negativibacillus | 1.80e-05 | -0.32% |
| f__Lachnospiraceae;g__[Eubacterium]_hallii_group | 0.014 | -0.32% |
| f__Anaeroplasmataceae;g__Anaeroplasma | 4.42e-06 | 0.30% |
| f__Christensenellaceae;g__Christensenellaceae_R-7_group | 3.36e-13 | 0.29% |
| f__Brevibacteriaceae;g__Brevibacterium | 5.54e-05 | 0.29% |
| f__Defluviitaleaceae;g__Defluviitaleaceae_UCG-011 | 1.46e-12 | 0.25% |
| f__Ruminococcaceae;g__ | 0.004 | 0.23% |
| f__Erysipelotrichaceae;g__uncultured_bacterium | 4.12e-13 | 0.21% |
| f__Ruminococcaceae;g__DTU089 | 0.004 | -0.18% |
| f__Lachnospiraceae;g__Lachnospiraceae_NK4A136_group | 0.002 | 0.17% |
| f__Ruminococcaceae;g__Oscillibacter | 7.52e-07 | -0.15% |
| f__Clostridiales_vadinBB60_group;g__ | 7.52e-07 | 0.15% |
| f__Eggerthellaceae;g__Gordonibacter | 1.03e-4 | -0.14% |
| f__Erysipelotrichaceae;g__ | 2.54e-09 | 0.13% |
| f__Bacteroidaceae;g__Bacteroides | 0.017 | -0.12% |
| f__Ruminococcaceae;g__Intestinimonas | 8.00e-05 | -0.10% |
| f__Dietziaceae;g__Dietzia | 1.71e-04 | 0.09% |
| f__Lachnospiraceae;g__Lachnospiraceae_UCG-006 | 6.83e-07 | 0.08% |
| f__Carnobacteriaceae;g__Jeotgalibaca | 0.020 | 0.08% |
| f__Ruminococcaceae;g__Anaerotruncus | 0.035 | -0.07% |
| f__Aerococcaceae;g__Aerosphaera | 0.003 | 0.06% |
| f__Peptococcaceae;g__uncultured | 8.45e-06 | 0.04% |
| f__Ruminococcaceae;g__Ruminococcus_2 | 0.030 | -0.04% |
| f__Lachnospiraceae;g__Lachnospiraceae_UCG-001 | 0.017 | -0.04% |
| f__Micrococcaceae;g__Glutamicibacter | 0.002 | 0.04% |
| f__Ruminococcaceae;g__Ruminococcus_1 | 1.92e-3 | 0.03% |
| f__Erysipelotrichaceae;g__Holdemania | 0.002 | -0.03% |
| f__Lachnospiraceae;g__Coprococcus_3 | 0.017 | 0.01% |

**Table S3**: **Pairwise comparison of phylogenetic diversity within housing conditions and between pens.** There was no significant difference in phylogenetic diversity within H1 between pens within the same feed intervention group (Kruskal Wallis, *p* > 0.05). In contrast, in H2 and H3 was a difference observed between pens within the same feed intervention (*p-*values bold).

| H1  PD | Pen1 | Pen2 | Pen3 | Pen4 | Pen5 | Pen6 | Pen7 | Pen8 | Pen9 |
| --- | --- | --- | --- | --- | --- | --- | --- | --- | --- |
| Pen2 | 0.934 | - | - | - | - | - | - | - | - |
| Pen3 | 0.708 | 0.473 | - | - | - | - | - | - | - |
| Pen4 | 0.786 | 1 | 0.936 | - | - | - | - | - | - |
| Pen5 | 0.862 | 0.626 | 0.339 | 0.408 | - | - | - | - | - |
| Pen6 | 1 | 0.786 | 0.473 | 0.626 | 0.936 | - | - | - | - |
| Pen7 | 1 | 1 | 0.786 | 0.786 | 0.708 | 0.862 | - | - | - |
| Pen8 | 0.339 | 0.626 | 0.936 | 0.786 | 0.234 | 0.234 | 0.551 | - | - |
| Pen9 | 0.551 | 0.551 | 0.234 | 0.339 | 1 | 0.708 | 0.473 | 0.284 | - |
| Pen10 | 0.862 | 1 | 0.551 | 0.551 | 0.862 | 1 | 0.862 | 0.473 | 0.862 |

| H2  PD | Pen1 | Pen10 | Pen2 | Pen3 | Pen4 | Pen5 | Pen6 | Pen7 | Pen8 |
| --- | --- | --- | --- | --- | --- | --- | --- | --- | --- |
| Pen10 | 0.031 | - | - | - | - | - | - | - | - |
| Pen2 | 0.031 | 0.786 | - | - | - | - | - | - | - |
| Pen3 | 0.284 | 0.083 | 0.146 | - | - | - | - | - | - |
| Pen4 | 0.002 | 0.551 | 1 | 0.002 | - | - | - | - | - |
| Pen5 | 0.626 | 0.009 | 0.021 | 0.061 | 0.002 | - | - | - | - |
| Pen6 | 0.031 | 0.862 | 0.786 | 0.044 | 0.626 | 0.009 | - | - | - |
| Pen7 | 1 | 0.061 | 0.044 | 0.473 | 0.021 | 1 | 0.044 | - | - |
| Pen8 | 0.083 | 0.626 | 0.936 | 0.147 | 0.862 | 0.021 | 0.626 | 0.031 | - |
| Pen9 | **0.002** | 0.339 | 0.339 | 0.473 | 0.014 | **0.003** | 0.188 | 0.284 | 0.284 |

| H3  PD | Iso1 | Iso10 | Iso2 | Iso3 | Iso4 | Iso5 | Iso6 | Iso7 | Iso8 |
| --- | --- | --- | --- | --- | --- | --- | --- | --- | --- |
| Iso10 | 0.408 | - | - | - | - | - | - | - | - |
| Iso2 | 0.626 | 0.862 | - | - | - | - | - | - | - |
| Iso3 | **0.005** | 0.083 | 0.147 | - | - | - | - | - | - |
| Iso4 | 0.044 | **0.031** | **0.031** | 0.002 | - | - | - | - | - |
| Iso5 | **0.011** | 0.188 | 0.234 | 0.147 | 0.002 | - | - | - | - |
| Iso6 | 0.339 | 0.113 | 0.284 | 0.003 | 0.284 | 0.002 | - | - | - |
| Iso7 | **0.002** | 0.002 | 0.003 | **0.002** | 0.002 | **0.002** | 0.002 | - | - |
| Iso8 | 0.284 | 1 | 0.936 | 0.014 | **0.002** | 0.014 | 0.061 | 0.002 | - |
| Iso9 | 0.473 | 0.786 | 1 | 0.054 | 0.083 | 0.147 | 0.284 | **0.002** | 1 |

**Table S4**: **Total number of genera or OTUs per pen, and the total number of genera or OTUs shared between pens.** Numbers are the mean for 7 individuals per pen.

| H1# genus | PEN1 | PEN2 | PEN3 | PEN4 | PEN5 | PEN6 | PEN7 | PEN8 | PEN9 | PEN10 |
| --- | --- | --- | --- | --- | --- | --- | --- | --- | --- | --- |
| PEN1 | **87** |  |  |  |  |  |  |  |  |  |
| PEN2 | 65 | **82** |  |  |  |  |  |  |  |  |
| PEN3 | 73 | 73 | **87** |  |  |  |  |  |  |  |
| PEN4 | 66 | 70 | 75 | **83** |  |  |  |  |  |  |
| PEN5 | 69 | 68 | 75 | 75 | **81** |  |  |  |  |  |
| PEN6 | 72 | 71 | 78 | 75 | 75 | **87** |  |  |  |  |
| PEN7 | 70 | 70 | 77 | 76 | 75 | 77 | **88** |  |  |  |
| PEN8 | 71 | 68 | 75 | 74 | 74 | 76 | 73 | **83** |  |  |
| PEN9 | 63 | 64 | 72 | 68 | 68 | 70 | 72 | 67 | **78** |  |
| PEN10 | 70 | 68 | 73 | 70 | 69 | 74 | 74 | 70 | 65 | **86** |

| H2#  genus | PEN1 | PEN2 | PEN3 | PEN4 | PEN5 | PEN6 | PEN7 | PEN8 | PEN9 | PEN10 |
| --- | --- | --- | --- | --- | --- | --- | --- | --- | --- | --- |
| PEN1 | **65** |  |  |  |  |  |  |  |  |  |
| PEN2 | 54 | **73** |  |  |  |  |  |  |  |  |
| PEN3 | 55 | 55 | **63** |  |  |  |  |  |  |  |
| PEN4 | 58 | 59 | 56 | **71** |  |  |  |  |  |  |
| PEN5 | 49 | 50 | 47 | 51 | **56** |  |  |  |  |  |
| PEN6 | 57 | 63 | 56 | 65 | 51 | **72** |  |  |  |  |
| PEN7 | 56 | 57 | 55 | 58 | 51 | 60 | **65** |  |  |  |
| PEN8 | 56 | 58 | 54 | 59 | 50 | 59 | 56 | **64** |  |  |
| PEN9 | 54 | 53 | 50 | 58 | 53 | 57 | 57 | 56 | **64** |  |
| PEN10 | 59 | 64 | 57 | 67 | 53 | 70 | 61 | 62 | 61 | **77** |

| H3  #genus | ISO1 | ISO2 | ISO3 | ISO4 | ISO5 | ISO6 | ISO7 | ISO8 | ISO9 | ISO10 |
| --- | --- | --- | --- | --- | --- | --- | --- | --- | --- | --- |
| ISO1 | **58** |  |  |  |  |  |  |  |  |  |
| ISO2 | 41 | **56** |  |  |  |  |  |  |  |  |
| ISO3 | 36 | 33 | **46** |  |  |  |  |  |  |  |
| ISO4 | 42 | 45 | 35 | **57** |  |  |  |  |  |  |
| ISO5 | 40 | 39 | 32 | 40 | **46** |  |  |  |  |  |
| ISO6 | 44 | 42 | 35 | 47 | 36 | **57** |  |  |  |  |
| ISO7 | 21 | 19 | 23 | 20 | 19 | 19 | **33** |  |  |  |
| ISO8 | 43 | 41 | 35 | 44 | 37 | 44 | 20 | **50** |  |  |
| ISO9 | 46 | 42 | 37 | 42 | 40 | 44 | 22 | 42 | **56** |  |
| ISO10 | 43 | 41 | 35 | 40 | 37 | 42 | 19 | 41 | 42 | **50** |

| H1  # OTU | PEN1 | PEN2 | PEN3 | PEN4 | PEN5 | PEN6 | PEN7 | PEN8 | PEN9 | PEN10 |
| --- | --- | --- | --- | --- | --- | --- | --- | --- | --- | --- |
| PEN1 | 416 |  |  |  |  |  |  |  |  |  |
| PEN2 | 239 | 392 |  |  |  |  |  |  |  |  |
| PEN3 | 263 | 275 | 436 |  |  |  |  |  |  |  |
| PEN4 | 243 | 246 | 267 | 373 |  |  |  |  |  |  |
| PEN5 | 233 | 240 | 256 | 252 | 367 |  |  |  |  |  |
| PEN6 | 230 | 256 | 269 | 242 | 233 | 394 |  |  |  |  |
| PEN7 | 234 | 252 | 277 | 235 | 248 | 242 | 434 |  |  |  |
| PEN8 | 258 | 241 | 247 | 244 | 239 | 233 | 229 | 384 |  |  |
| PEN9 | 222 | 258 | 271 | 232 | 238 | 250 | 264 | 222 | 397 |  |
| PEN10 | 263 | 268 | 274 | 254 | 231 | 245 | 252 | 245 | 247 | 436 |

| H2  # OTU | PEN1 | PEN2 | PEN3 | PEN4 | PEN5 | PEN6 | PEN7 | PEN8 | PEN9 | PEN10 |
| --- | --- | --- | --- | --- | --- | --- | --- | --- | --- | --- |
| PEN1 | **265** |  |  |  |  |  |  |  |  |  |
| PEN2 | 171 | **309** |  |  |  |  |  |  |  |  |
| PEN3 | 181 | 188 | **278** |  |  |  |  |  |  |  |
| PEN4 | 156 | 204 | 186 | **331** |  |  |  |  |  |  |
| PEN5 | 154 | 185 | 169 | 168 | **253** |  |  |  |  |  |
| PEN6 | 171 | 212 | 186 | 199 | 178 | **303** |  |  |  |  |
| PEN7 | 170 | 185 | 173 | 169 | 174 | 190 | **274** |  |  |  |
| PEN8 | 173 | 205 | 181 | 184 | 176 | 217 | 194 | **307** |  |  |
| PEN9 | 164 | 194 | 174 | 183 | 189 | 211 | 189 | 207 | **318** |  |
| PEN10 | 176 | 219 | 196 | 219 | 187 | 237 | 195 | 231 | 222 | **343** |

| H3  # OTU | ISO1 | ISO_2 | ISO_3 | ISO_4 | ISO_5 | ISO_6 | ISO_7 | ISO_8 | ISO_9 | ISO_10 |
| --- | --- | --- | --- | --- | --- | --- | --- | --- | --- | --- |
| ISO_1 | **212** |  |  |  |  |  |  |  |  |  |
| ISO_2 | 114 | **209** |  |  |  |  |  |  |  |  |
| ISO_3 | 80 | 71 | **132** |  |  |  |  |  |  |  |
| ISO_4 | 109 | 121 | 72 | **216** |  |  |  |  |  |  |
| ISO_5 | 90 | 86 | 55 | 81 | **168** |  |  |  |  |  |
| ISO_6 | 98 | 99 | 64 | 113 | 72 | **224** |  |  |  |  |
| ISO_7 | 32 | 27 | 41 | 27 | 25 | 27 | **123** |  |  |  |
| ISO_8 | 101 | 100 | 69 | 115 | 77 | 102 | 25 | **203** |  |  |
| ISO_9 | 98 | 88 | 74 | 80 | 77 | 77 | 32 | 81 | **183** |  |
| ISO_10 | 100 | 103 | 68 | 102 | 74 | 85 | 25 | 101 | 81 | **197** |

**Table S5: Effect of dietary treatment and housing condition on mean concentrations of acetate, butyrate, propionate and lactate.**

| Item |  | Acetate | Butyrate | Lactate | Propionate |
| --- | --- | --- | --- | --- | --- |
| Housing condition | Dietary treatment |  |  |  |  |
| H1 | -MCFA | 7.03 (1.45) | 1.74 (0.44) | 0.10 (0.09) | 1.04 (0.23) |
|  | +MCFA | 6.60 (1.39) | 1.44 (0.39) | 0.16 (0.01) | 0.77 (0.19) |
| H2 | -MCFA | 9.01 (1.42) | 2.40 (0.67) | 0.92 (0.29) | 0.48 (0.06) |
|  | +MCFA | 9.21 (0.99) | 2.79 (1.23) | 1.00 (0.26) | 0.67 (010) |
| H3 | -MCFA | 6.90 (2.15) | 1.20 (0.34) | 0.76 (0.15) | 0.54 (0.37) |
|  | +MCFA | 7.84 (1.72) | 1.38 (0.35) | 0.84 (0.28) | 0.48 (0.09) |
| Housing condition |  |  |  |  |  |
| H1 |  | 6.81^a^ (1.36) | 1.59^a^ (0.42) | 0.13^a^ (0.07) | 0.90^a^ (0.25) |
| H2 |  | 9.11^b^ (1.16) | 2.60^b^ (0.96) | 0.96^b^ (0.27) | 0.58^b^ (0.13) |
| H3 |  | 7.37^a^ (1.90) | 1.29^a^ (0.30) | 0.80^b^ (0.22) | 0.52^b^ (0.26) |
| Dietary treatment |  |  |  |  |  |
| -MCFA |  | 7.65 (1.87) | 1.78 (0.69) | 0.60(0.40) | 0.69(0.35) |
| +MCFA |  | 7.89 (1.70) | 1.87 (0.97) | 0.67(0.43) | 0.64(0.17) |
| *P*-value |  |  |  |  |  |
| Dietary treatment |  | 0.680 | 0.718 | 0.372 | 0.503 |
| Housing condition |  | 0.008 | <0.001 | <0.001 | <0.001 |
| Housing condition x dietary treatment | | 0.623 | 0.485 | 0.989 | 0.067 |
|  |  |  |  |  |  |

Each group consisted of 5 pens with 7 male broilers. Means in the same column with common superscripts differ (*P adj* <0.05), SD between brackets.

**Table S6: Effect of dietary treatment and housing condition on mean body weight, average daily gain, feed intake, and gain to feed ratio.**

| Item |  | BW 35 d,g | 0-14  ADG | ADFI | G:F | 14-35 ADG | ADFI | G:F | 0-35  ADG | ADFI | G:F |
| --- | --- | --- | --- | --- | --- | --- | --- | --- | --- | --- | --- |
| Housing condition | Dietary treatment |  |  |  |  |  |  |  |  |  |  |
| H1 | -MCFA | 2094 (110) | 27.2 (1.6) | 31.5 (1.8) | 0.865 (0.015) | 78.7 (4.1) | 119.3 (5.7) | 0.660 (0.005) | 57.5 (2.8) | 83.2 (3.4) | 0.692 (0.006) |
|  | +MCFA | 1955 (80) | 24.8 (1.7) | 28.7 (1.6) | 0.864 (0.016) | 73.8 (2.9) | 107.6 (3.4) | 0.686 (0.017) | 53.4 (2.3) | 74.7 (2.8) | 0.715 (0.008) |
| H2 | -MCFA | 2331 (126) | 26.5 (1.4) | 30.4 (1.1) | 0.870 (0.048) | 89.3 (5.1) | 121.1 (5.5) | 0.737 (0.017) | 62.5 (3.8) | 82.4 (4.3) | 0.758 (0.018) |
|  | +MCFA | 2275 (55) | 26.8 (0.9) | 31.5 (0.4) | 0.848 (0.026) | 86.0 (2.0) | 116.9 (8.7) | 0.738 (0.044) | 60.8 (1.4) | 80.5 (4.7) | 0.757 (0.038) |
| H3 | -MCFA | 2421 (130) | 28.9 (2.4) | 37.0 (2.9) | 0.781 (0.032) | 91.5 (4.2) | 129.8 (10.4) | 0.707 (0.038) | 64.5 (3.7) | 89.7 (7.0) | 0.720 (0.034) |
|  | +MCFA | 2324 (237) | 29.6 (3.1) | 36.4 (2.9) | 0.813 (0.058) | 90.8^*^(6.0) | 130.1^*^(4.9) | 0.693^*^(0.031) | 65.1^*^(4.1) | 91.6^*^(2.6) | 0.710^*^(0.031) |
| Housing condition |  |  |  |  |  |  |  |  |  |  |  |
| H1 |  | 2025^a^ (117) | 26.0^a^ (2.0) | 30.1^a^ (2.1) | 0.864^a^ (0.014) | 76.3^a^ (4.2) | 113.5^a^ (7.6) | 0.673^a^ (0.017) | 55.5^a^ (3.2) | 78.9^a^ (5.4) | 0.703^a^ (0.013) |
| H2 |  | 2303^b^ (97) | 26.6^a^ (1.1) | 36.7^b^ (1.0) | 0.797^b^ (0.047) | 87.7^b^ (4.0) | 119.0^a^ (7.2) | 0.738^b^ (0.031) | 61.6^b^ (2.8) | 81.5^a^ (4.3) | 0.757^b^ (0.027) |
| H3 |  | 2373^b^ (188) | 29.2^b^ (2.6) | 31.0^a^ (2.8) | 0.859^a^ (0.038) | 90.6^b*^(4.8) | 130.0^b*^(7.9) | 0.698^a*^(0.034) | 64.2^b*^(3.6) | 90.3^b*^(5.2) | 0.712^a*^(0.031) |
| Dietary treatment |  |  |  |  |  |  |  |  |  |  |  |
| -MCFA |  | 2283 (182) | 27.5 (2.0) | 32.9 (3.5) | 0.838 (0.053) | 86.5 (7.1) | 123.4 (8.4) | 0.701 (0.04) | 61.5 (4.4) | 85.1 (5.9) | 0.723 (0.035) |
| +MCFA |  | 2185 (218) | 27.1 (2.8) | 32.2 (3.7) | 0.842 (0.041) | 83.0^*^(8.1) | 117.6^*^(11.2) | 0.707^*^(0.04) | 59.4^*^(5.5) | 81.6^*^(7.8) | 0.728^*^(0.034) |
| *P*-value |  |  |  |  |  |  |  |  |  |  |  |
| Dietary treatment |  | 0.0602 | 0.529 | 0.328 | 0.837 | 0.036 | 0.035 | 0.599 | 0.080 | 0.056 | 0.720 |
| Housing condition |  | <0.001 | 0.003 | <0.001 | <0.001 | <0.001 | <0.001 | <0.001 | <0.001 | <0.001 | 0.002 |
| Housing condition x dietary treatment | | 0.7937 | 0.179 | 0.179 | 0.116 | 0.559 | 0.151 | 0.312 | 0.267 | 0.057 | 0.180 |
|  |  |  |  |  |  |  |  |  |  |  |  |

Each group consisted of 5 pens with 15 male broilers in H1 and 8 male broilers in H2 and H3. Means in the same column with common superscripts across housing condition differ (*P adj* <0.05), SD between brackets. BW= body weight. ADG= Average daily gain. ADFI= the average daily feed intake. G:F=gain to feed ratio. *one isolator is excluded in those analyses because of management problems.
